# Spatiotemporal variations in b-value suggest an evolving mechanical state of the crust in the southeastern Alps

**DOI:** 10.1038/s41598-026-51916-x

**Published:** 2026-05-20

**Authors:** M. Picozzi, D. Spallarossa, D. Bindi

**Affiliations:** 1 National Institute of Oceanography and Applied Geophysics – OGS, Trieste, Italy; 2 Department of Physics ‘Ettore Pancini’, Università di Napoli Federico II, Naples, Italy; 3 DISTAV, University of Genoa, Genoa, Italy; 4 Helmholtz Centre Potsdam, GFZ German Research Centre for Geosciences, Potsdam, Germany

**Keywords:** Natural hazards, Solid Earth sciences

## Abstract

Understanding whether and how the mechanical state of the crust evolves is a central challenge in intraplate regions. We investigate the southeastern Alps region by analyzing the frequency-magnitude distribution of ten years of seismicity (14776 earthquakes) with magnitudes in the range 0 ≤ M_L_ ≤ 4.5. We analyze the spatio-temporal evolution of the b-value, a proxy for crustal stress and strength. Using the b-positive method and Singular Spectrum Analysis, we observe a persistent b-value decrease since 2020 in the Friuli region, which we interpret as evidence of the evolving stress state of the system towards crustal weakening. . By linking b-value trends to the Hoek–Brown failure criterion, we interpret the crustal stress evolution in terms of rock mass disturbance. Our results suggest that, even under low loading rates, distinct crustal volumes may evolve differently over time, with some potentially approaching critical conditions while others remain stable. Whitin this framework, b-value variations may provide a physically grounded approach to monitor fault weakening and assess seismic hazard in slowly deforming continental regions.

**Supplementary Information:**

The online version contains supplementary material available at 10.1038/s41598-026-51916-x.

## Introduction

Understanding crustal strength evolution is a key objective that may provide hints for intercepting long-term preparatory processes toward future moderate-to-large earthquakes^1^. For several years now we have been implementing in different regions of Italy the principles of Seeber and Armbruster^2^, who suggested using minor and micro-earthquakes as proxies for the mechanical state of the crust (e.g.^3–11^). Here, we attempt to answer the question of whether monitoring the relative event size distribution (b-value) and, in particular, its spatio-temporal evolution can allow us to infer crustal strength and capture hints of the preparatory phase of large earthquakes.

While seismologists and engineers primarily use the magnitude–frequency distribution (MFD) and the b-value^12^ for Probabilistic Seismic Hazard Assessment (PSHA) studies^13^, it is worth noting that widely cited studies have shown that lower b-values are often associated with higher differential stress, both in laboratory and tectonic settings, providing the conceptual basis for interpreting b-value decreases in terms of evolving crustal conditions^14,15^. Previous studies have also interpreted temporal decreases in b-value as possible indicators of preparation toward failure or mechanically evolving fault systems (e.g.^16–18^) For example, Nanjo et al.^17^ reported a decade-scale decrease in b-value prior to the 2011 Tohoku and 2004 Sumatra great earthquakes and interpreted this as evidence of increasing stress toward large rupture. Nanjo et al.^17^ revealed b-value changes in and around the focal areas of the M6.9 and M6.8 earthquakes that occurred off the Pacific coast of Miyagi prefecture, northeastern Japan, on March 20 and May 1, 2021. Concerning Italy, Sugan et al.^19^ documented a decrease in b-value prior to the 2009 L’Aquila mainshock, followed by an increase during the aftershock phase, consistent with a transition from stress accumulation to stress release. These studies support the interpretation of b-value as a sensitive proxy of the evolving mechanical state of the crust. In volcanic systems, the b-value is commonly interpreted as a proxy for the evolving mechanical state of the crust, integrating the effects of stress, temperature, and fluid migration. At Campi Flegrei, for instance, b-value variations have been shown to delineate rheological heterogeneities within the caldera, with low values marking high-stress, mechanically stronger regions (e.g., caprock), and high values identifying fluid-rich, highly fractured domains^20^. In addition, damage-based and laboratory-inspired frameworks have linked changes in earthquake size distributions to progressive damage accumulation and localization, offering a mechanistic context in which decreasing b-values may accompany weakening^21^.

The new physical way to interpret b value evolution as proxy of crustal stress in relation to the occurrence of earthquakes at various spatial and temporal scales matches with the availability of high-resolution earthquake catalogs through which have a new look at the processes leading to rupture both at natural and laboratory scales (e.g., among many^22^in laboratory^6,19,23–25^ for natural earthquakes^26,27^ for the forecasting of strong aftershocks).

Spatial and temporal changes in the b-value are thus commonly interpreted as reflecting variations in the stress field and are increasingly applied to monitor the preparatory phases of large earthquakes. Notably, a consistent decrease in b-value is now frequently detected within the epicentral region weeks to months before major events, suggesting progressive stress accumulation and fault system evolution (e.g^19,24,28,29^).

**Fig. 1:**
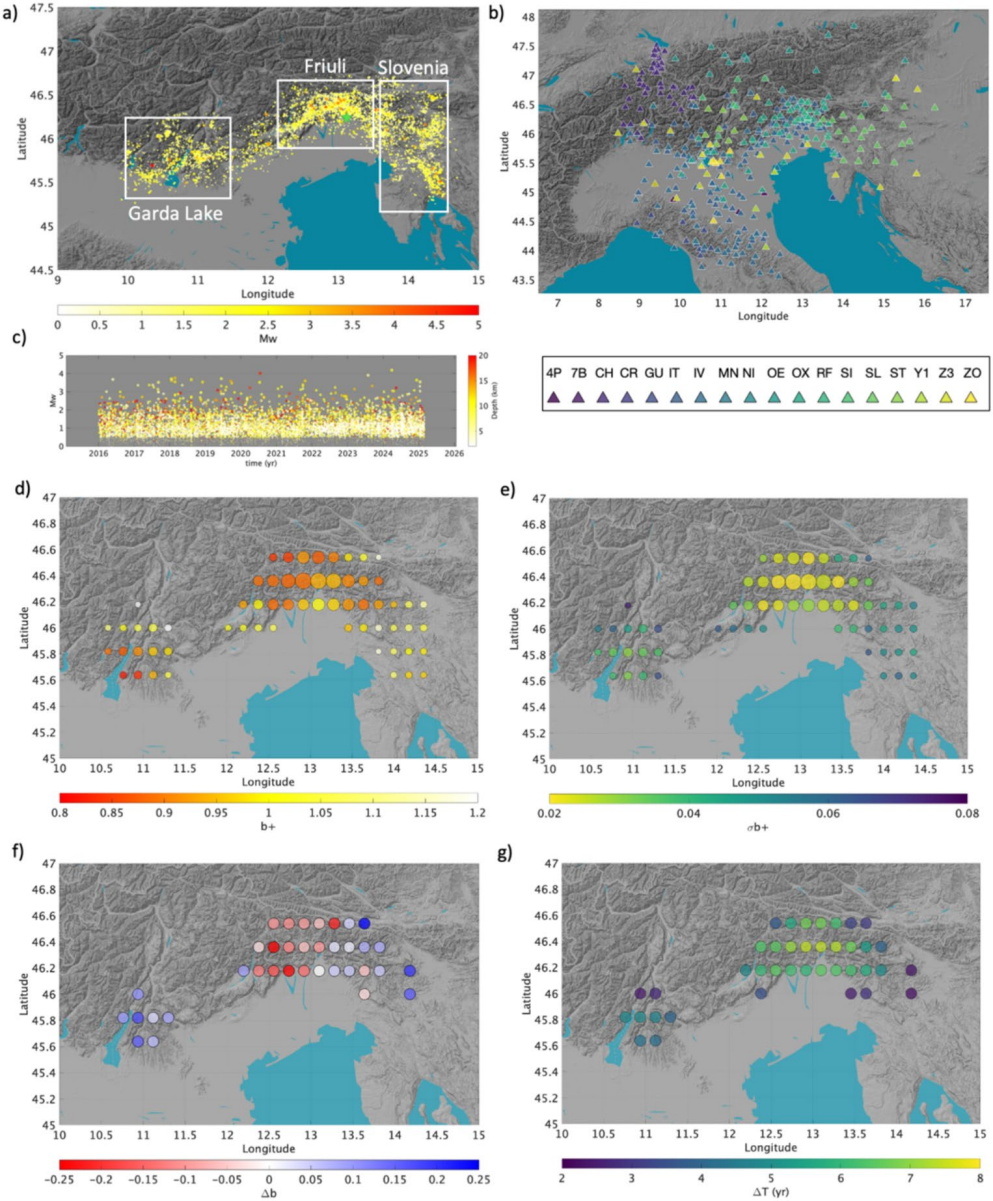
(**a**) Distribution of earthquakes colored per Mw. (**b**) Map showing the seismic stations (green triangles; see section “Data availability and resources” for the DOI associated to each network). (**c**) Temporal evolution of seismicity considering Mw and with data colored per hypocentral depth. (**d**) Map showing mean of b-value from bootstrap for background seismicity over a regular grid. The dimension of dots is related to the amount of data for grid node. (**e**) Map showing uncertainty of b-value from bootstrap analysis for the background seismicity over a regular grid. (**f**) Map of Δb for background seismicity for the period of study 2016–2025. Only $\Delta \mathrm{b}$ values with $\mathrm{p}<0.05$ are displayed. (**g**) Duration of the b-value time series from which is estimated Δb.

For the Italian and regional civil protection monitoring duty, we focus our study on the southeastern European Alps, known tectonically as the Adriatic Indenter. This region results from the interaction between the Eurasian plate and the Adriatic microplate^30,31^. The area exhibits a variety of tectonic styles, including compressive faults, strike-slip systems and, to a small extent, extensional structures. The historical seismicity is characterized by the occurrence of moderate to strong seismicity, which includes: the ‘1117 Verona Earthquake’ (Me 6.8) in the Venetian plain^32^, the ‘1348 Villach Earthquake’ (Me 6.6) near the border between Italy, Slovenia and Austria^33^, the ‘1695 Asolo Earthquake’ (Mw 6.5) along the southernmost alpine fronts^34^, the ‘1511 Idrija Earthquake’ (Mw ~6.8) linked to the Dinaric strike-slip fault system (Fitzko et al., 2005), and the more recent ‘1976 Friuli Earthquake’ (Mw 6.5,Fig. 1) caused by thrust faulting along the southern Alps front^35^. The geodetic monitoring highlights a persistent crustal shortening and strain accumulation at rates of ~2–10 × 10⁻⁸ yr⁻1, placing the region among the most actively deforming continental intraplate zones in Central Europe^36^.

We adapted the Rapid Assessment of Seismic Moment and Radiated Energy (RAMONES) procedures developed by Spallarossa et al.^37^ for central Italy to the seismic monitoring of the southeastern Alps. We reprocessed ten years of seismicity and analyzed 14776 earthquakes with magnitudes in the range 0 ≤ M_L_ ≤ 4.5, as recorded by permanent seismic networks (Fig. 1a, see Data and Resources). After declustering and applying a data quality selection, we computed the seismic moment (M_0_) for 9438 earthquakes, which in turn have been used to estimate the b-value. We implemented the b-positive approach^38^, which is insensitive to transient changes in catalog completeness and allows for robust b-value estimation.It is worth mentioning that implementing a fully automated and robust processing strategy, RAMONES allow one to derive M_0_, which is then used to derive Mw^39^, even for small earthquakes. The direct estimation of Mw independently from scaling relation with M_L_, as traditionally occurs for small earthquakes, avoids the potential biases in the estimation of b-value due to changes in stress drop (Δσ) and apparent velocity, which hinder M_L_-Mw scaling relations (as discussed by^40^. Furthermore, we acknowledge that our observations are made possible by the efforts of the seismological community to increase the seismic network density and develop data mining strategies to obtain robust, augmented seismic catalogs (e.g.^37^).

In the following, we describe the estimation of the b-value from background seismicity and its spatio-temporal evolution. To interpret the b-value on a physical basis, we link it to the Hoek–Brown failure criterion^41^.

### b-value spatial distribution in southeastern Alps

We reprocessed the dataset of earthquakes available from the OGS bulletin (https://www.crs.inogs.it/bollettino_new/; see section Data availability and resources), which occurred in the period 2016.01.01–2025.02.27 and recorded by 445 velocimetric and accelerometric stations from the seismic networks monitoring the area (Fig. 1b). After estimating M_0_ by tuning the RAMONES procedure for the southeastern Alps region (Fig.S1, see method), we computed the moment magnitude Mw^39^,Fig. 1c). Before analyzing the earthquake frequency-magnitude distribution, we separated the events into background and clustered seismicity (Fig. S2) using a nearest-neighbor method^42^. This preliminary analysis is important because the background seismicity reflects the long-term tectonic loading and stress conditions, whereas clustered seismicity (e.g., aftershocks and swarms) is typically driven by short-term, transient processes (e.g., stress redistribution, pore-pressure diffusion, and dynamic triggering). Including clusters without correction may bias the b-value and obscure tectonic signals. Picozzi et al.^24^ discussed the importance of discriminating between the two kinds of seismicity when studying the preparatory phase of the 2009 L’Aquila earthquake.

The background seismicity (Fig. S2d) appears to be distributed across the entire monitored area, although the Friuli region (east of longitude 12°) exhibits higher seismic activity than the western sector. Even the clustered seismicity appears unevenly distributed (Fig. S2e), and in agreement with the results by Petersen et al.^43^. Worth of being noted, the latter appears mostly concentrated in the Friuli region too, where eight over fifteen large historical earthquakes (Mw >= 6) occurred^44^, Figure S3a). Notably, even in recent years, the largest events (Mw 3) have been recorded in the same area (Fig. S3b). Observing the seismicity spread over the Friuli area well complies with the idea of considering the crust as a fractured, heterogeneous *rock mass* made of blocks, faults, joints, and zones of damage^45^. This interpretation is consistent with studies showing that the seismicity of the south-eastern Alps is influenced by a complex stress field, as well as by the interaction of multiple fault systems (e.g.^46,47^).For estimating the b-value, we have followed the same approach of previous studies where this parameter was exploited to investigate the crustal stress evolution^6,24,25,48,49^ (Palo et al.2023 see Methods). To avoid biases in estimating b-value due to the combination of seismicity generated by different processes, the b-value analysis is performed separately on the two subsets (i.e., background and clustered seismicity).Hereinafter, we focus our discussion on the b-value for the background seismicity. We performed the b-value analysis on background seismicity only because clustered events are dominated by cascade triggering and by short-term aftershock incompleteness, which may distort the observed frequency-magnitude distribution (FMD) and generate apparent b-value changes unrelated to the long-term regional stress state. By declustering the catalog, we aim to isolate the component of seismicity that more faithfully reflects the persistent tectonic loading and thus is more appropriate for investigating temporal changes in crustal conditions.

**Fig. 2:**
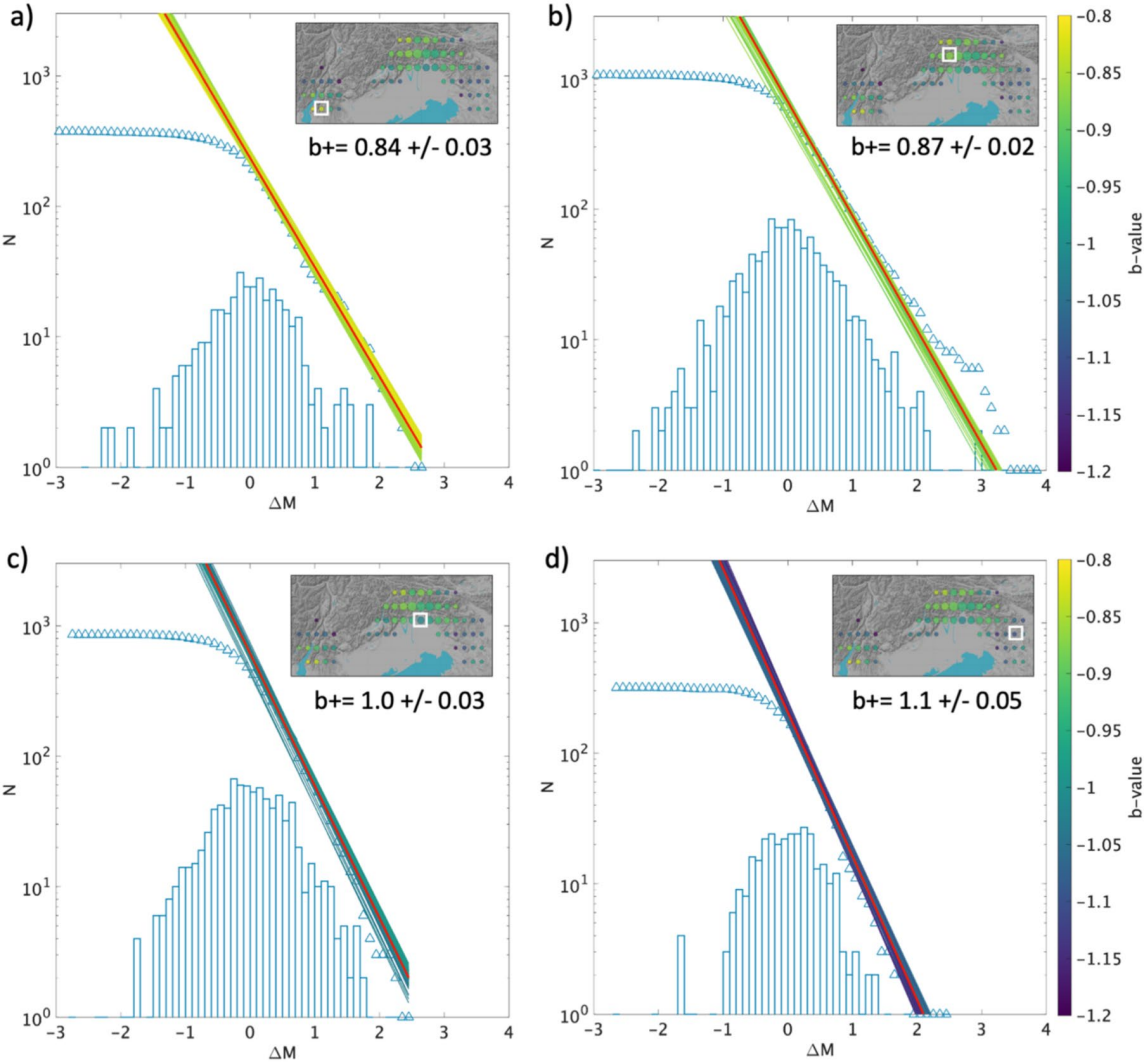
Panels (**a–d**) show examples of frequency magnitude distributions, FMDs, for ΔM (histogram), its cumulative frequency distribution (triangles) and the Gutenberg-Richter model with b-positive value from the bootstrap distribution in the range estimation mean value +/- 1 standard deviation (colored lines according to the b-positive value) and the one for the mean b-positive value (red line). Insets display the geographic location of each node. The figures for all other nodes are uploaded as supplemental material.

Similarly to Picozzi et al.^50^, we map the spatial variability of b-value (see Methods) over a regular grid with a 20 km step and considering earthquakes with depth between 2.5 and 15 km, which encompasses the seismogenic volume where large earthquakes are generated (Fig. 1d). We then compute the b-value for each grid node, considering the seismicity within a 25 km hypocentral distance from each node (Table S3). This distance is compatible with the rupture length of the 1976 Friuli earthquake. Only 62 grid nodes have at least 200 events within a 25 km radius (Fig. S4). Specifically, the number of events associated to each node varies between 201 and 1432 (Figure S5). Four examples of FMDs are shown in Fig. 2 for different sectors of the investigated area (the FMDs for all nodes are made available separately, see Data Availability and Resources).

The uncertainty associated with the parameter is estimated using a bootstrap approach^51^ by repeating the analysis for each grid node generating 500 random sampling realizations of the original data set with replacement^52^.

Our results show an heterogenous spatial distribution of b-value through the area, with higher values (b+>1) in the Slovenian sector, and smaller ones (b+ <1) both in the Friuli and Garda Lake areas (Figs. 1d and 2). The low standard error associated with the b-value (Fig. 1e) confirms the robustness of the observations. We even explored the spatial distribution of b-value testing different numbers of events per node (Fig. S6) and a different grid (Fig. S7). Even varying these parameters, we can observe that main features of the b-value spatial distribution are preserved, confirming the robustness of results.

From a seismic hazard perspective, the most concerning areas are precisely those in which b-value is less than 1. Lower b-values are observed in the Friuli sector, west of the 1976, Mw 6.5 Friuli Earthquake (Fig. 1). It is important to note that this area hosts well known seismogenic sources (i.e., Maniago-Sequals, Andres-Forgaria nel Friuli, Tramonti-Montemaggiore, DISS Working Group, 2018 – version 3.2.1) which have the potential of generating large earthquakes with Mw between 5.9 and 6.5.

We also performed the analysis for the clustered seismicity (Fig. S8). Due to our strict criteria for computing b-value, we only performed the analysis for 18 grid nodes in the Friuli area. This is not surprising since the clustered seismicity is mostly located in that area, as mentioned before. The b-value for the clustered seismicity is low (most of grid nodes show b-value close to 0.8), which is consistent with previous studies. For example, Gentili & Bressan^53^ show that aftershocks in northeastern Italy and western Slovenia are characterized by b-values ranging from 0.8 to 1.1. These values reflect a relatively low b-value environment, which is consistent with high stress and compressional tectonics.

### b-value spatio-temporal evolution

Significant research has been conducted on how the b-value evolves over time, primarily via sliding-window statistical methods (e.g., laboratory experiments^54^:,natural earthquakes^24,27^: and 2023b, induced seismicity in geothermal fields^6,48^. To our knowledge, no study has attempted yet to separate trends, oscillatory components, and noise within b-value time-series. To this end, we implemented a non-parametric signal decomposition approach, the Singular Spectrum Analysis^55^, and we analyzed the b-value time series generated for the grid node covering the area of study. In this study, we focus on trends in b-value, because we are mainly interested in long-term crustal processes that influence a large portion of the crust and can potentially lead to large earthquakes. Conversely, even small stress changes induced by tidal loading have been found to be able to modulate b-value^56^. Hence, future studies will further investigate this issue in the region under study.

To compute the b-value time series, we require that the node have at least 300 events within 25 km. As before, the b-value is computed on sets of 200 events with a 10-event window shift. For each time window, the b-value refers to the origin time of the last event. Figure (S9a) shows the comparison between the b-value time series considering a window shift of one sample (gray squares) and ten samples (red dots). The trends derived by the SSA analysis (Fig. S9b) show that using a different window shift does not affect the stability of the trends. The selection of a window shift of 10 events is preferred for computational convenience. These criteria led to the estimation of the b-value time series for 39 nodes (Fig. S4b).

**Fig. 3:**
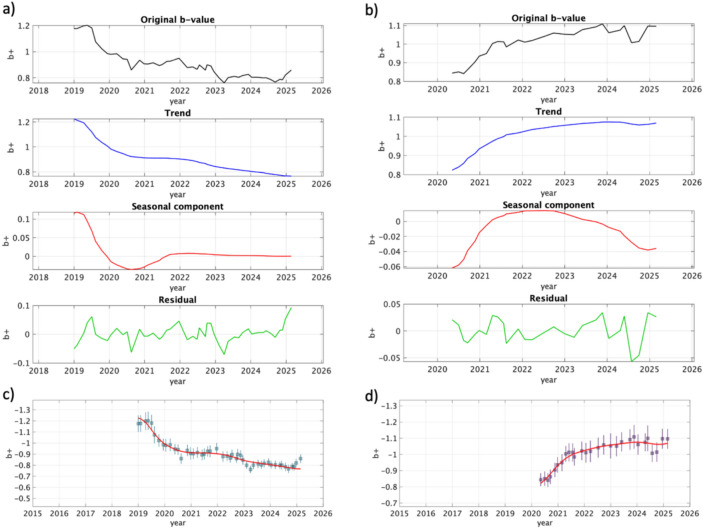
Examples of SSA Analysis on b-value time-series of two grid nodes (Figures for all the other nodes are available as supplemental material).(**a**) and (**b**) examples of original b-value time-series (black), trend (blue), seasonal component (red), and residuals (green). (**c**) and (**d**), comparison between original b-value time-series (light blue squares) with uncertainty (blue vertical lines) and trend component (red).

We found b-value time series that increased and decreased over time. Two examples are shown in Fig. 3, while the figure showing all the nodes is made available separately (see Data Availability and Resources). The SSA analysis separates each time series into three components: trend, oscillatory component, and residual (Fig. 3a, b). Hence, the SSA decomposition help us to separate the b-value time series into components that can be interpreted as reflecting distinct physical processes: a long-term trend associated with progressive stress accumulation and damage evolution, an oscillatory component that captures transient forcing such as fluid-driven or seasonal modulation of effective stress, and a residual term that represents stochastic fluctuations arising from small-scale heterogeneities and short-term seismic variability. Here we focus on the multi-year trend, interpreted as an expression of the long-term mechanical evolution of the system, consistent with the established relationship between b-value and stress. The oscillatory component, which may reflect external hydrogeological forcing, is beyond the scope of this study and will be explored in future research. The SSA analysis is carried out using the maximum theoretically allowed window length (L), which is set to 15. Figure (S10) shows that, even when the parameter L is varied over a wide range between 10 and 20, the separability of the elementary signal components is preserved and the results are stable.

Focusing on the trend component, which closely resembles the original time series (Fig. 3c, d), we investigate how b-values vary over time and space. We modeled the extracted trend components using Gaussian Process Regression (GPR). GPR was applied to the extracted trend to obtain a smooth and continuous representation, alongside with uncertainty estimates. Therefore, SSA acts as a data-driven filter, while GPR provides a probabilistic interpolation of the filtered signal.

To test for significant differences in mean b-values between periods, we applied a z-test using the mean and standard deviation values derived from both time intervals. The resulting z-score and p-value indicate whether the change in b-value is statistically significant.

We estimated the b-value difference, Δb, for each grid node over the entire monitoring period (Fig. 1f). For each time series, we calculated Δb by considering the average b-value for the first year (b_0_) and the average b-value for the last year (b_f_) (i.e., Δb = b_f_ -b_0_; Table S4). The duration of the b-value time series is shown in Fig. 1g.

Significant Δb values were found for all 39 nodes except one. Interestingly, negative Δb were found in the Friuli area, reaching as low as −0.25 (Fig. 1f). In contrast, Δb values increased (up to +0.25) in both the Garda Lake and Slovenia regions.

Figure 7 combines yearly maps of absolute b-values (panels a-h) with the corresponding Δb anomalies relative to a reference period (panels i-p). This allows us to distinguish the spatial distribution of seismicity scaling from its temporal evolution, highlighting coherent multi-year changes in the mechanical state of the crust. The spatiotemporal evolution of b-values reveals a progressive decrease in the Friuli sector, with increasingly negative Δb anomalies after 2020, suggesting a coherent regional change in the mechanical state of the crust.According to Scholz^18^, b-value variations can be interpreted in terms of crustal differential stress. Positive b-value changes can then be related to lower differential stress in the crust, potentially due to an increase in pore pressure. Bachmann et al.^46^ identified this mechanism in relation to induced earthquakes during an Enhanced Geothermal System (EGS) experiment in Basel, Switzerland. Similar direct observations are difficult to obtain in the case of natural seismicity. In the area under study, Bressan et al.^57^ observed the seismicity to cluster along areas with sharp transitions in crack density and mechanical/structural heterogeneity. It is hypothesised that these transition zones are characterized by high heterogeneity due to overlapping tectonic phases, which may also reflect variable pore pressures and reduced confining stress.Conversely, studies have reported a decrease in b-values before the 2009 Mw 6.1 L’Aquila earthquake^19^ and the 2023 Mw 7.8 Kahramanmaras earthquake in Türkiye^25,28^. Interestingly, the low Δb area compares well to the high fractal dimension values found by Bressan et al.^47^. They also found that the spatially distributed seismicity is associated with rather complex earthquake sequences^58^. Comparing the spatial distribution of clustered seismicity with the temporal evolution of the b-value reveals a marked spatial coherence between these observables (i.e., Fig. 1e vs. Fig. S2e and Fig. S8a). Clusters are predominantly concentrated in the Friuli sector, which also exhibits the strongest negative Δb anomalies. This spatial correspondence suggests that seismic clustering develops preferentially in regions undergoing a progressive shift towards lower b-values, which are commonly interpreted as being indicative of increasing differential stress and fault loading. This interpretation is consistent with recent high-resolution observations of microseismicity in the Eastern Alps. Analysis of the Swath-D large-N network highlights a pronounced spatial zonation of seismicity, with the highest activity and the largest number of seismic sequences concentrated along the southeastern Alpine front, including the Friuli region^43^. In this area, seismicity is dominated by numerous short-lived sequences that often exhibit mainshock–aftershock characteristics and are associated with thrust faulting under N–S to NNW–SSE compression. Notably, these regions of intense clustered seismic activity correspond to areas of strong crustal attenuation and inferred high fracture density. This suggests the presence of mechanically weak and stressed crustal volumes^43^.

### Crustal strength evolution inferred through b-value

The presence of diffuse seismicity, low b-value with clear evidence of temporal change in the Friuli area southeastern Alps during the 2016-2025 interseismic period, prompted us to seek a strategy for interpreting our b-value observations in terms of rock damage production (e.g., as done by^59^ for California,and^60^ for southern Italy). Notably, Núnez-Jara et al.^61^recently observed a progressive rock weakening during the preparation phase of the 2023, Mw 7.8 Kahramanmaras, Türkiye. Spatial and temporal variations in b-value have been widely used to infer stress heterogeneity and its evolution within the framework of Coulomb failure (e.g.^62,63^). The use of Coulomb-based interpretations in seismology inherently relies on several simplifying assumptions, such as the validity of an effective frictional failure criterion, near-critical stress conditions, and the use of seismic observables as indirect proxies of the stress state. These assumptions are common to a wide class of studies and define the conceptual framework within which our results should be interpreted.Rather than adopting the classical Mohr–Coulomb failure criterion, we use the Hoek–Brown (HB) formulation^41^, which provides a more appropriate description of the mechanical behavior of fractured and heterogeneous rock masses. While the Coulomb criterion assumes linear strength envelopes and constant material properties, the HB approach explicitly accounts for the progressive degradation of rock strength through parameters such as the Geological Strength Index and the disturbance factor D. This makes it particularly suitable for describing damaged crustal volumes, such as Friuli, where deformation is distributed and evolves over time, as supported by geomechanical and seismological observations^57^. In this context, the use of the HB criterion allows us to incorporate the effects of damage accumulation inferred from seismic observations within a physically consistent framework.

The disturbance factor (D) in the HB criterion allows to account for the degree of damage or disturbance in a rock mass (i.e., it varies from 0 in case of undisturbed rock to 1 for very disturbed rock). While originally defined to capture engineering-induced disturbance (e.g., stress relief around excavations or blasting damage), it can also be interpreted as a proxy for natural damage in tectonic or faulted settings. Increasing D leads to a progressive reduction of cohesion and frictional resistance of the rock mass.

In our case, linking b-value variations to the rock mass disturbance (D) enables a physically consistent interpretation of the spatio-temporal evolution of seismicity. Specifically, we link Δb to D through a logistic function (see Methods for a summary of HB).

**Fig. 4:**
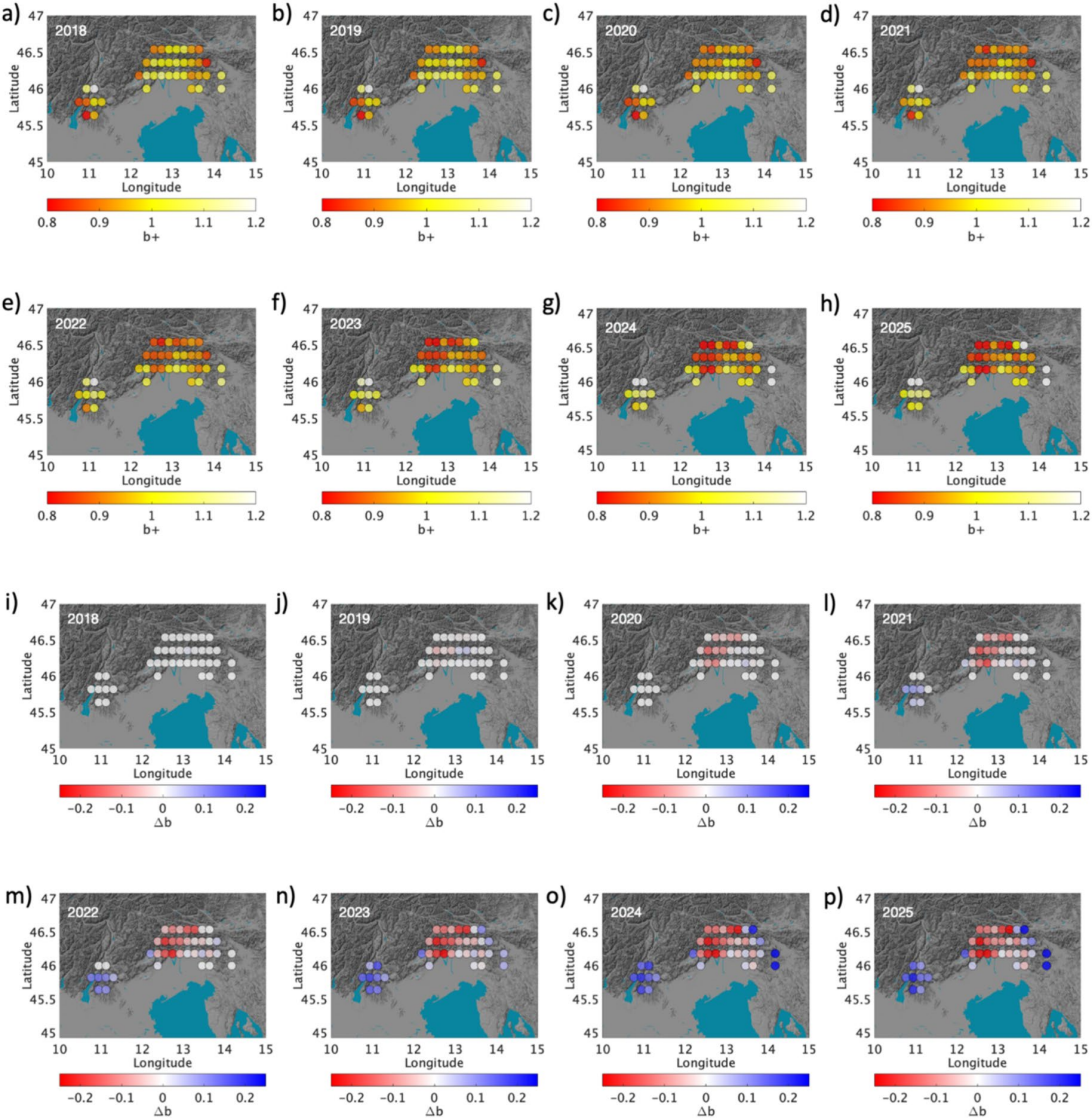
Panels (**a–h**), annual maps of b-values estimated on a regular spatial grid; (**i–p**) corresponding changes (Δb) relative to the initial reference year. Colors indicate b-value magnitude (top panels) and temporal variations (bottom panels), highlighting a progressive decrease in b-value in the Friuli sector over time, spatially coincident with clustered seismicity.

Worth of note, besides the disturbance parameter, HB also considers the Geological Strength Index (GSI) and the material property of the intact rock (m_i_) as strength parameters (Fig. 4). In this study, however, we link Δb to the rock mass disturbance only, while the values for GSI and m_i_ are kept constant. The rationale behind this choice is that we assume m_i_ (depending on the petrography of the intact rock) and GSI (describing the macroscopic structure of the rock mass, as: joint geometry, fracturing) reflecting the stable lithological and structural nature of the carbonate crust in northeastern Italy over decadal timescales. Conversely, we allow D to vary in time to account for progressive rock mass damage driven by distributed microseismicity, localized stress accumulation, and aseismic deformation. This approach enables us to capture crustal strength evolution acting on a decadal timescale without invoking changes in lithology or macro-structural setting. We acknowledge that this mapping is model-dependent and does not provide a unique or direct measurement of fault properties. Instead, D should be interpreted as a physically motivated proxy that describes the relative evolution of crustal damage and strength.

We show in Fig. 4 a schematic image of the Mohr diagram for effective stress conditions considered plausible for the area under study at seismogenic depth (see Methods). Furthermore, we show in the figure the HB failure criterion where the set of HB parameters has been selected to be compatible with a weak, fractured rock mass (see Methods), while the rock mass disturbance factor D has been varied between 0 (undisturbed) and 1 (very disturbed).

As discussed, the link between Δb and D is established via a logistic function (Fig. 4b). This choice is not arbitrary but reflects a set of physically motivated constraints: i) the disturbance factor D is bounded between 0 and 1, requiring a saturating mapping; ii) small variations in Δb are expected to produce limited changes in D when the medium is either weakly or highly damaged, implying reduced sensitivity at the extremes; and iii) the transition between these regimes is inherently nonlinear, with maximum sensitivity at intermediate damage levels. The logistic function provides the simplest monotonic formulation that satisfies these requirements, ensuring a smooth, bounded, and scale-controlled transformation between Δb and D.

The logistic mapping between Δb and the disturbance factor D is defined by a single parameter, *k*, which controls its steepness and thus acts as a gain factor. Varying *k* within a reasonable range (5–15; Fig. S11-S15) modifies only the amplitude of D variations, without affecting the relative spatial or temporal patterns, similarly to how the friction coefficient μ in the Mohr–Coulomb criterion shifts stress thresholds without altering stress distribution. Consequently, the identification of areas of increasing or decreasing disturbance remains robust (Fig. S12-S15), and *k* = 10 is adopted as a balanced reference value.

**Fig. 5:**
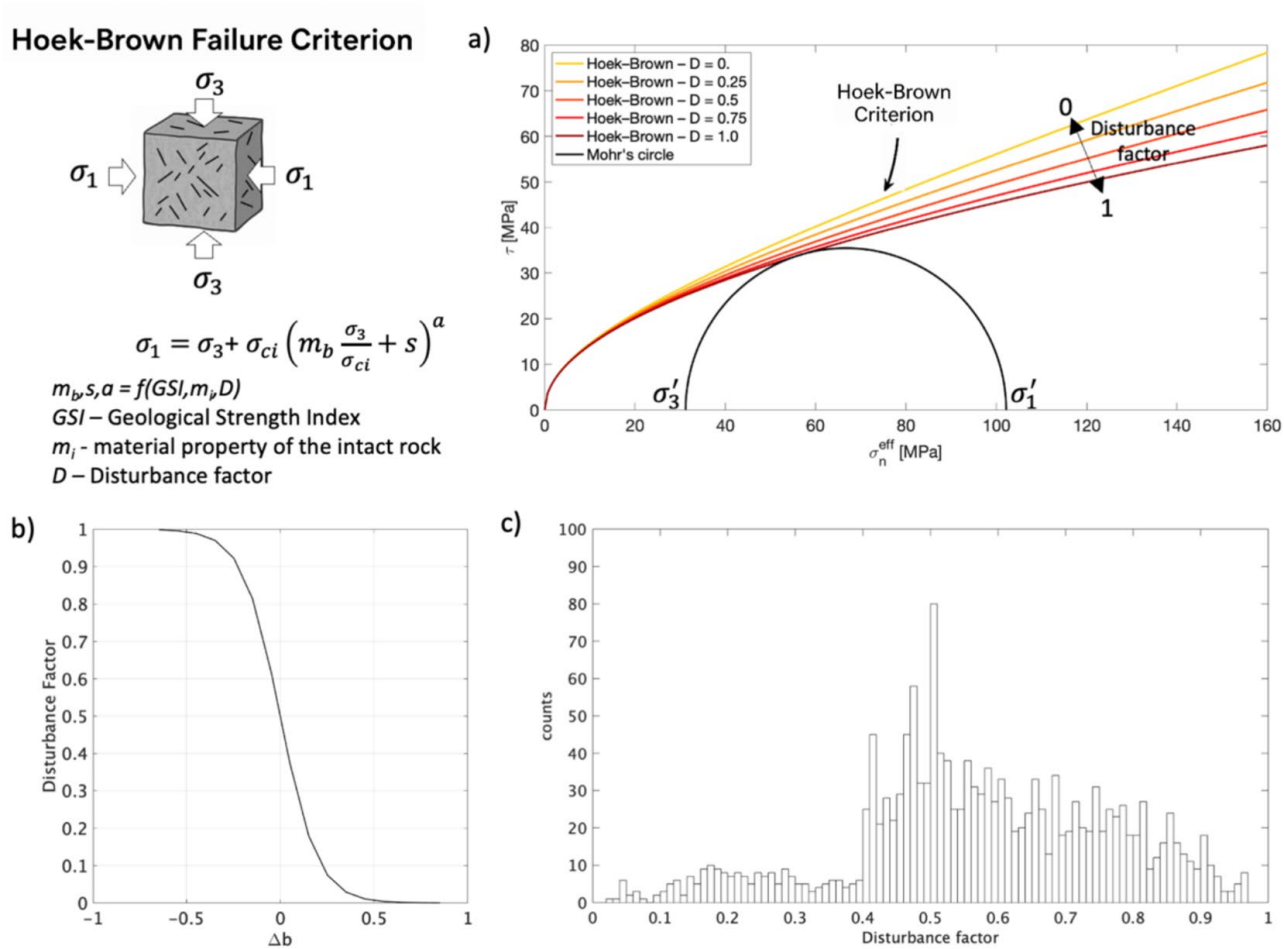
Outline of the Hoek-Brown failure criterion. (**a**) HB criterion τ-σ_n_ curves for D between 0 (undisturbed, yellow) and 1 (disturbed, brown). Mohr’s circle (black line). (**b**) Logistic function relating Δb to the disturbance factor. (**c**) Distribution of the disturbance factor for Δb obtained over time for each grid node.

The D values derived by considering how Δb varies over time for each grid node are mostly greater than 0.5 (Fig. 4c). According to our criterion, this indicates an increase in rock mass disturbance. To illustrate the spatial and temporal evolution of D more clearly, we repeated the analysis for each grid node every year (Fig. 5). The disturbance factor exhibits a clear spatiotemporal evolution, with a progressive increase in the Friuli sector beginning around 2020 and intensifying through 2025. This pattern is spatially coherent and is largely confined to the central-eastern portion of the study area. In contrast, adjacent regions show lower and more stable values, suggesting a localized evolution of crustal damage.

### Interpretations of the crustal strength evolution

Our analyses reveal a robust, multi-year decrease in the b-value in the Friuli sector, spatially coincident with enhanced clustered seismicity. Specifically, the b-value persists over a multi-year timescale rather than appearing as a short-lived fluctuation. Similar temporal patterns have previously been reported in association with evolving stress conditions (e.g.^17^^,^^61^) providing a physically grounded framework for interpretation. Overall, the convergence of evidence supports the interpretation in terms of crustal weakening, although alternative explanations cannot be ruled out.

Translating the b-value into the disturbance factor D enables us to incorporate our interpretation of the results into a rock-mechanics framework (as is often done with the Mohr–Coulomb model). We therefore interpret the progressive increase in the disturbance factor D observed in the Friuli area since 2020 as indicative of the evolving damage state of the crust. This trend is consistent with increasing microcrack density, potential pore-pressure effects, and localized stress accumulation along pre-existing fault structures, providing an independent and physically grounded perspective that supports the proposed interpretation. Our results suggest a transition toward a stronger stress concentration regime and localized faults weakening^14,18^. The spatial expansion of D, that over time involves multiple nodes, implies that damage is not confined to a single fault but is propagating through a wide, distributed damage zone. These trends might be consistent with the observations in other tectonic settings (e.g.^19,61,64^) where diffuse damage accumulation preceded major ruptures. Our observations may be due to ongoing tectonic loading along the Alpine-Dinaric front, fluid overpressure migration, or the activation of secondary structures within the carbonate-dominated lithologies of Friuli, or a combination of these factors.

Importantly, such an evolution does not necessarily imply that the system is progressing towards a large earthquake. Alternative scenarios should be considered, such as those involving a long-lived, partially self-regulated deformation process. For instance, the accumulation of damage and an increase in D could lead to enhanced distributed deformation, whereby strain is accommodated through repeated moderate-magnitude events or aseismic processes rather than culminating in a major rupture. Within this framework, microcrack development and fluid-assisted weakening could promote stress redistribution and transient weakening without necessarily reaching a critical instability. In slowly deforming regions such as Friuli, characterized by low loading rates, such processes may persist over extended timescales. This allows the system to evolve through episodic or oscillatory behavior rather than a monotonic progression towards failure. Therefore, while our observations are consistent with a mechanically evolving crust and potentially increasing stress concentration, they may also reflect a stable or metastable regime in which deformation is accommodated without leading to a large earthquake^65^. Furthermore, we clarify that our interpretation remains at the level of hypothesis and should be tested in the future against independent observables, such as geodetic strain, stress-drop or radiated-energy proxies, relocation-based structural evolution, or multiparametric indicators of the crustal state.

**Fig. 6:**
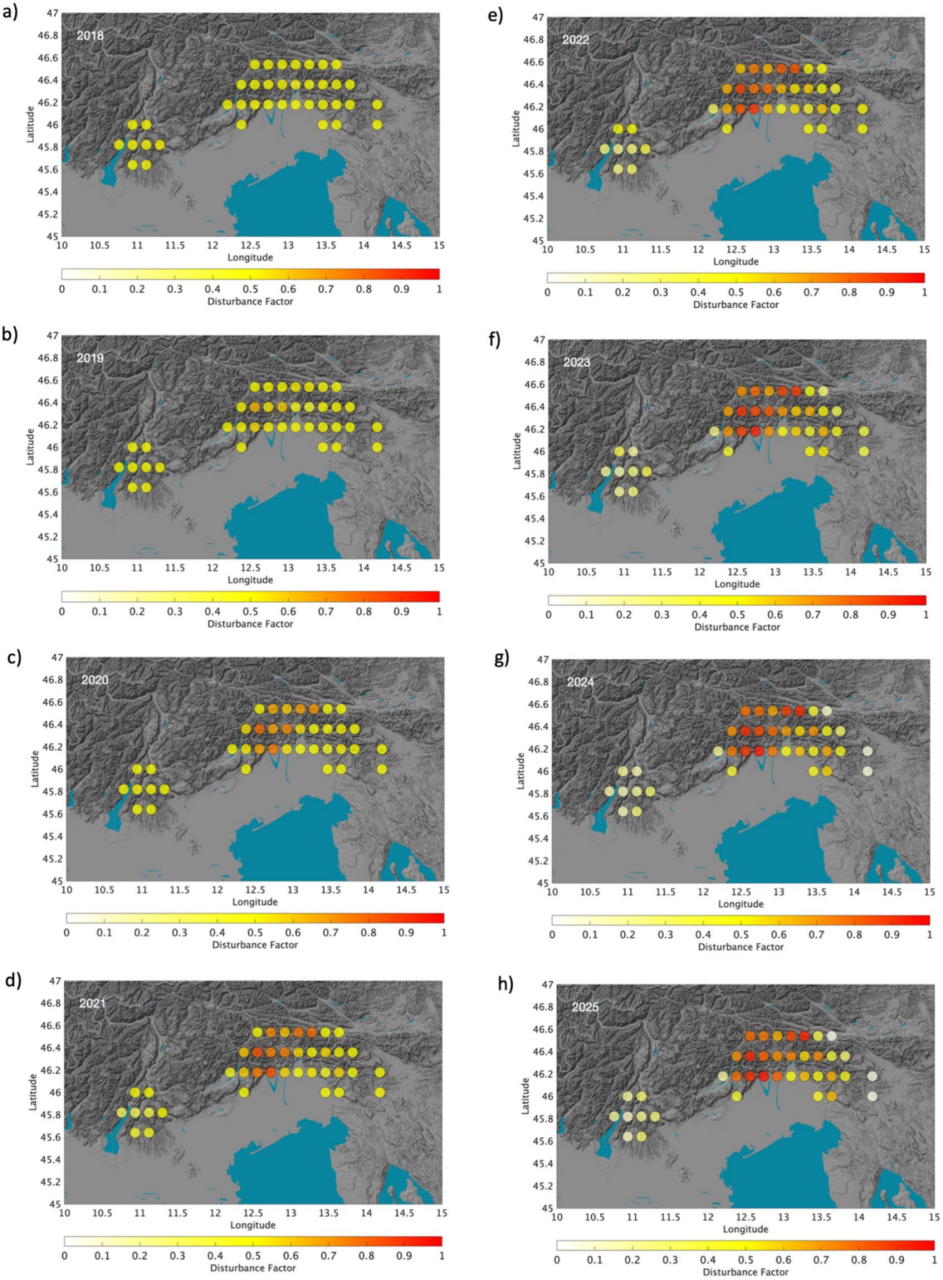
Spatio-temporal evolution of the disturbance factor. From (**a**) to (**h**), maps showing D over a regular grid.

Following Scholz^18^, we translate the b-value temporal evolution into variations in shear stress resistance τ_res_(t) (Fig. 6). For estimating the shear stress resistance τ_res_, we reparametrized the Scholz’s relation (2015) between b-value and the differential stress (σ_D_) using only the Italian dataset (Fig. S16, see Methods). Thus, the empirically derived increase (decrease) in b-value is thus translated into a temporal decrease (increase) in peak shear strength. Changes in τ_res_ can vary by up to ~10 MPa over the observation period (~6 years) (Fig. 6a). These are first-order estimates and the absolute values would change when different parameters are selected. However, the overall trend would be preserved. We consider these values reasonable and of the same order of magnitude as the stress drop observed by Franceschina et al.^66^ and Cataldi et al.^3,67^. As illustrated in Fig. 6, our results reveal contrasting behaviors in neighboring regions. The Friuli area exhibits progressive, spatially coherent crustal evolution which suggests a weakening process, which contrasts with minimal disturbance (or even an increase in resistance) in its western and eastern regions (Fig. 6b, c). Notably, the moment rate for Friuli and Garda Lake regions is comparable. This indicates that, despite being affected by similar seismic activities, the crustal response to the loading rate occurring in these two areas is distinct. As previously mentioned, the Friuli sector corresponds to the area with the highest number of large historical earthquakes^44^. The coexistence of enhanced clustering and negative Δb anomalies in Friuli suggests a fault system evolving under sustained, albeit minor, tectonic loading.Here, stress accumulation promotes both the temporal organization of seismicity into sequences, as well as a systematic shift in earthquake size statistics. This is in contrast to regions such as the northwestern Alps, where swarm-like activity and higher b-values are observed. This further supports the link between clustering style, magnitude–frequency distribution, and underlying mechanical conditions. The observations of our study highlight the need for more in-depth seismic hazard studies in the southeastern Alps area. However, it is also important to remember that low b-values alone are not deterministic precursors, and that the fault system will not necessarily fail within a specific timeframe.

Because of the low loading rate in the area (~1–2 mm/yr^68^;, the low b-value can persist for several years without culminating in a large earthquake, as stress may be released through moderate earthquakes (Mw 4.5–5.5) or aseismic creep, prolonging the interseismic phase. Our results demonstrate that small earthquakes provide essential information for refining short-term seismic hazard models in structurally complex regions that exhibit crustal evolution. The proposed modeling approach links observed trends in precursory seismicity (e.g., a decreasing b-value) to a quantitative reduction in rock mass shear resistance, offering insights into whether a fault system is approaching criticality. This study represents the first step toward developing a virtual model that integrates seismological, geochemical, and geological data with computational simulations, enabling replication of the physical and dynamic processes of the fault systems in the southeastern Alps.

## Data & methods

### Data

We analyze 14776 earthquakes with magnitude in the range 0 ≤ M_L_ ≤ 4.5 available from the OGS bulletin (https://www.crs.inogs.it/bollettino_new/). The earthquakes occurred between 1 January 2016 and 10 January 2025 and were recorded by 445 velocimetric and accelerometric stations belonging to the permanent and temporary seismic networks in the southeastern Alps.

Waveforms processing is done following the procedure of Spallarossa et al.^37^ and includes : instrumental correction and automated picking as proposed by Scafidi et al.^69^,event re-location using the NonLinLoc software^70^,estimation of the local magnitude M_L_ by averaging station magnitude estimates and considering the zero-magnitude attenuation model calibrated by Di Bona^71^ for Italy but without applying station corrections, to ensure homogeneous magnitude estimates across all events. The earthquake location is carried out using the 1D regional velocity model^72^ as in the OGS localizations and Cataldi et al.^3^. The resulting locations are consistent with those of the OGS bulletin, with uncertainties in event locations mostly within 1 km horizontally (Erh) and 2 km vertically (Erz).

From this data set, we select a subset of 9,438 earthquakes that satisfies the following quality criteria: events localized using at least 12 phases between P- and S-waves; events with Erh ≤ 3 km and Erz ≤ 5 km; events with an azimuthal gap smaller than 200°.

## Seismic moment

Following the RAMONES processing workflow developed by^73^, (Picozzi et al.2019b) and Spallarossa et al.^37^ and Picozzi et al.^50^, we compute the seismic moment M_0_ from S-wave peak displacement (PD_S_) measured on waveforms. In the first step, we consider the seismic moment M_0_ of 681 earthquakes obtained by Cataldi et al.^3^ using GITpy^74^, and we analyze their waveforms to estimate, for each recording, the S-wave peak displacement (PD_S_). These earthquakes are distributed over the whole region of interest (Figure S1a), the hypocentral depths are mostly included between 5 km and 15 km (Figure S1b), and the magnitudes cover the range Mw 1.5 to Mw 4 (Figure S1c). We follow the Scafidi et al.^69^: a high-pass corner frequency of the pre-deconvolution filter is automatically determined based on signal-to-noise analysis, selecting the lowest frequency in the 0.3–2.0 Hz range for which the signal-to-noise ratio is larger than 4.0.

We compute the PD_S_ considering a time window starting 0.1 s before the S-wave onset and ending at different percentages of the cumulated energy varying with the source to site distance R: (i) 90% when R < 25 km; (ii) 80% when 25 km < R < 50 km; (iii) 70% when R > 50 km. We imposed a minimum time window length of 2.5 s and a maximum time window length of 20 s for PD_S_. For each recording, we measure the signal-to-noise ratio considering a pre-event noise window of the same length as the direct S-waves. The estimates from the NS and EW components are averaged (geometric mean).

The PD_S_ values relevant to the kth earthquake recorded at the ith station are then linked to M_0_ through the empirical attenuation model:

1 $$log{\left[{PD}_{S}\left({R}_{H}\right)\right]}_{ki}=A+B\cdot log{\left({M}_{0}\right)}_{k}+{w}_{j}{C}_{j}+\left(1-{w}_{j}\right){C}_{j+1}+{\sum}_{i=1}^{{N}_{sta}}{\delta}_{il}{S}_{i}$$

where, the hypocentral distance (R_H_) range is discretized into Nbin; the index j = 1,.., Nbin indicates the jth node selected such that R_H_ is between the distances rj ≤ R_H_ < rj+1; the attenuation function is linearized between nodes rj and rj+1 using the weights w, computed as wj = (rj+1 – RH)/(rj+1 – rj). The distance range 10–120 km is discretized into 50 bins equally spaced on a logarithmic scale. The Si terms are the station corrections for station i, and Nsta is the number of stations, where we constrain the station corrections to zero mean. The coefficients A, B, and Cj are determined by solving the over-determined linear systems (Eq. 1) in a least-square sense.

To fix the trade-off between A and Cj, the attenuation is constrained to zero at R equal to 10 km. The calculated regressions have a R-squared correlation coefficient (R^2^) equal to 0.87.

The attenuation model ${C}_{j}$ for southeastern Alps agrees with the one estimated for Central Italy by Spallarossa et al.^37^, (Fig. S1d).

The station corrections *S*_*i*_ are shown in Fig. S1e. Both the station corrections and the coefficients of the attenuation models are reported in Table S1 and S2 (Supplemental Material). Figure S1f shows the comparison between the M_0_ from Cataldi et al.^3^, with the values obtained correcting PD_S_ for attenuation effects.

The procedure is then applied to 9,438 earthquakes to estimate the seismic moment, M_0_ by correcting the PD_S_ values for each event for attenuation and site and obtaining M_0_ through Eq. (1).

### Generalized Hoek–Brown criterion

We model the progressive weakening of the fault zone adopting the Hoek–Brown failure criterion, HB, a non-linear empirical relation widely used to describe the strength of fractured rock masses^41^. It relates the major and minor principal stresses at failure through parameters calibrated on intact rock strength, the rock mass quality (GSI) and the disturbance factor (D), which quantifies mechanical damage due to tectonics or fluid activity.

Therefore, unlike the classic Mohr–Coulomb model, which assumes constant cohesion and friction angle, the Hoek–Brown criterion allows capturing the strength degradation caused by in-situ fracturing and damage accumulation through the disturbance factor (D).

The evolution of the HB parameters allows us to describe how the rock mass shear resistance decreases over time as structural damage increases, consistent with the mechanical behavior observed in carbonate fault zones.

2 $${\sigma}_{1}={\sigma}_{3}+{\sigma}_{ci}\cdot {\left({m}_{b}\cdot \frac{{\sigma}_{3}}{{\sigma}_{ci}}+s\right)}^{a}$$

where ${\sigma}_{ci}$ is the unconfined compressive strength of intact rock, mb, s and a are empirical constants derived from the intact material parameter mi, Geological Strength Index (GSI), and disturbance factor, D. The parameter D ranges between 0 and 1 describing the undisturbed and fully disturbed rock mass conditions, respectively.

The HB criterion is commonly adopted for studying rock masses in geotechnical applications, but we believe it also can provide a useful representation of crustal failure processes in seismically active carbonates such as those of southeastern Alps.

For estimating the shear stress resistance τ_res_, we first reparametrized the relation between b-value and the differential stress (σ_D_ = σ_1_ - σ_3_) following^18^, but using only the Italian dataset, as follows:

3 $$\sigma_D = a\cdot b + c$$

where, *a* is equal to −431.7 and *c* is 545.6 (Fig. S16).

Eq.(3) is used to compute σ_D_ for each grid node considering the first estimation of b-value for each time series (b_0_). Then, according to the tectonic of the area, we assume σ_3_ as vertical, and we consider 40° as average dip value for the seismogenic sources in the area of study (i.e., Maniago-Sequals, Andres-Forgaria nel Friuli, Tramonti-Montemaggiore, DISS Working Group, 2018 – version 3.2.1) and we compute σ_1_ and σ_3_. Then, we obtain the effective stresses σ_1_’ and σ_3_’ considering the pore fluid pressure at depth as hydrostatic and we compute τ_res_.

### b-value

Van der Elst^38^ introduced a novel method based on the frequency distribution of magnitude difference, termed the b-positive method. This innovative method enable more robust b-value estimations, even in scenarios where the magnitude of completeness (Mc) may vary spatially and temporally.

We follow Mitsui et al.^52^ for the implementation of the approach.

The b-positive method leverages the magnitude difference, symbolized as M′, instead of relying on the magnitude M. Specifically, it relies on magnitude increments (M′ ≥ *d*M’) observed between consecutive earthquakes. We set *d*M’ equal to 0.2 magnitude units. This approach culminates in the following equation:

4 $$f\left({M}{\prime}\right)=\beta \cdot exp \left(-\beta {M}{\prime}\right)$$

Eq.(2) is based on the mathematical principle that the difference between parameters following an exponential distribution (here represented by Eq. 2) adheres to a Laplace distribution (double exponential distribution). Then, b-value is estimated following relation

5 $$b= \frac{{log}_{10}(e)}{\overset{\_}{{M}{\prime}}-(dM-0.05)}$$

where M′ represents the average magnitude increment and 0.05 is the correction constant to compensate for bias in magnitude estimates, which are rounded off to the nearest 0.1 (i.e., the catalog’s resolution). We derive the uncertainty following a bootstrap approach^51^, and repeating the analysis for each grid node with 500 random sampling realizations of the original data set with replacement.

We follow Picozzi et al.^7^ for the spatial analysis. We define a regular grid with a 20 km step. For each node, we select events with distances within 25 km and depth between 2.5 and 15 km (which encompasses the seismogenic volume where large earthquakes are generated) and estimate the b-value. Nodes that have less than 200 events within 25 km are discarded. Nodes considered in the b-value analysis have a number of event varying between 201 and 1432 (Figure S5).

The b-value estimates together with the uncertainty and the number of events for each node are reported as Supplemental Material (Table S3).

In the temporal analyses, we require that a grid node has at least 300 events within 25 km, and we estimate the b-value considering a moving window of 200 events that shift of 10 events at time, similarly to Picozzi and Iaccarino^6^ and Picozzi et al.^25^. For each time window, the b value is associated to the origin time of the last event.

Also in this case, we estimate the uncertainty following a bootstrap approach^51^ with 500 random sampling realizations. We compare b-value differences, Δb, between two time periods considering their standard deviations

6 $$z= \frac{({b}_{1}-{b}_{2})}{\sqrt{{\sigma}_{1}^{2}+{\sigma}_{2}^{2}}}$$

Therefore, we can reject the null hypothesis that two b-values come from the same populations at the 95% confidence level if z exceeds 1.96.

**Fig. 7:**
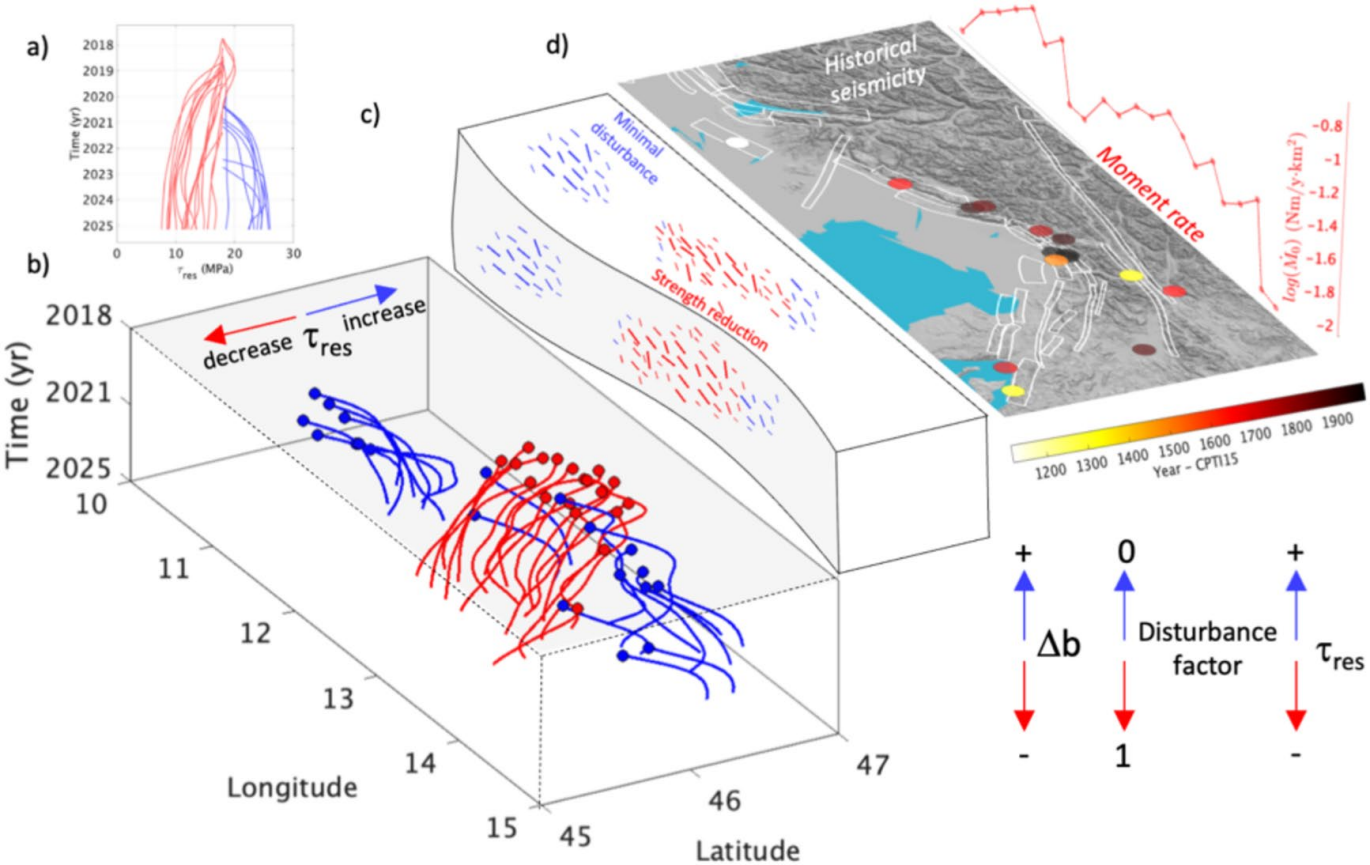
Outline summarizing the unified observations. (**a**) Temporal evolution of shear resistance grid nodes with negative (red) and positive (blue) Δb(t) estimates. (**b**) 3D representation (Latitude, Longitude, Time) of Temporal evolution of shear resistance grid nodes with negative (red) and positive (blue) Δb(t) estimates. (**c**) Southeastern Alps with highlighted the crustal sector characterized by strength reduction (red) and those with minimal disturbance (blue). (**d**) Distribution of historical seismicity (all events with magnitude Mw >= 5.9 in the time range 1000-2020, taken from the Italian Parametric Earthquake Catalogue, CPTI15^44^; and colored per year of occurrence. Seismogenic sources from DISS (white boxes,https://diss.ingv.it) The logarithm of moment rate for the seismicity occurred in the period 2016- 2025 is shown in red.

Finally, we link Δb to D through a logistic function

7 $$D\left(b\right)=\frac{1}{1+exp(-k\cdot\Delta b)}$$

where Δb is equal to b_i_ – b_0_, b0 is the b-value for the reference period (i.e., typically the average for the first year of the time series), bi refer to the testing period (i.e., the average b-value for a given year), and k is the steepness (i.e., it controls how sensitive D is to changes in b). We verified the impact of the parameter k on our D estimates. The D maps obtained using k equal to 8 and 12 (Fig. S10) show minor changes in D values for grid nodes but the same trend of the D map shown in the main text and obtained using k equal to 10 (Fig. 7).

### Singular spectrum analysis, SSA

We implement the statistical non-parametric method SSA^55^ to analyze the b-value time-series. SSA is particularly powerful because it does not require an explicit model of the data, since it consists of a principal component technique in the time domain that relies on the singular valued decomposition of a specific matrix constructed upon the time-series (i.e., it is a special Hankel matrix built from the original time series, and its role is to transform the 1-D time series into a multidimensional representation that reveals patterns such as trends and periodicities). Thus, SSA decomposes the original series into a sum of a small number of interpretable components such as a slowly varying trend, oscillatory components and noise (e.g., see^75^, for an application to InSAR data).

In this study, we extend the SSA analysis to the b-value time series, and we decompose them into three components using the maximum theoretically allowed window length (L). After some tuning, we found that large L values offered better separability of the elementary signal components. Thus, we set the L equal to half of each time-series length. We interpret the first component as trend, the second as oscillatory component, and the last as noise.

The extracted trend component was then modeled using Gaussian Process Regression (GPR). The initial noise variance was set to half the standard deviation of the original data, and predictors were standardized to improve numerical stability. This approach allowed for smooth interpolation of the trend and provided credible intervals for uncertainty quantification.

### Clustering by nearest-neighbor distance, η

We applied the nearest-neighbor approach^76^, which computes the generalized distance between pairs of earthquakes, η, by an analysis of the time-space distances between pairs of earthquakes. The parameter η is obtained by estimating the distances in time (i.e., rescaled time, Tη) and space (i.e., rescaled distance, Rη) between an event i and its parent j, where both distances are normalized by the magnitude of the parent event. The rescaled time and distance are computed as follows:

8 $${T}_{ij}={t}_{ij}{10}^{-b\frac{{m}_{i}}{2}}$$

9 $${R}_{ij}={\left({r}_{ij}\right)}^{{D}_{c}}{10}^{-b\frac{{m}_{i}}{2}}$$

where, m is the magnitude, b is the parameter of the Gutenberg–Richter law, which plays the role of exponential weight of the earlier event i by its magnitude, and Dc is the fractal dimension. Finally, η is defined as:

10 $$log{ (\eta }_{ij})= log({R}_{ij})+ log({T}_{ij})$$

We compute η considering the epicentral location of the earthquakes. According to Zaliapin and Ben-Zion^77^, we set the b equal to 0 to mitigate the presence of artifacts due to the overlap of earthquakes’ domain of attraction with background seismicity, and we use Dc equal to 1.5.

We model the η distribution with a sum of a log-Gaussian function and we use the threshold value $log{ (\eta }_{0})=$ 4.2 to split the earthquakes population in clustered (C) and background (B) seismicity.

## Supplementary Information

Supplementary Information.

## Data Availability

The initial seismic catalogue used in this paper, all the processed data, and the figures showing for each node the b-value estimates and time-series are available at the ZENODO repository 10.5281/zenodo.17157398]. Other data and software are available on request. The data used in this work was provided by the Dfollowing networks: [CH] Swiss Seismological Service (SED) At ETH Zurich. (1983). National Seismic Networks of Switzerland. ETH Zürich. 10.12686/sed/networks/ch [IT] Presidency of Council of Ministers—Civil Protection Department. (1972). Italian Strong Motion Network [Data set]. International Federation of Digital Seismograph Networks. 10.7914/SN/IT [IV] Istituto Nazionale di Geofisica e Vulcanologia (INGV). (2005). Rete Sismica Nazionale (RSN) [Data set]. Istituto Nazionale di Geofisica e Vulcanologia (INGV). 10.13127/sd/x0fxnh7qfy [MN] MedNet Project Partner Institutions. (1990). Mediterranean Very Broadband Seismographic Network (MedNet) [Data set]. Istituto Nazionale di Geofisica e Vulcanologia (INGV). 10.13127/sd/fbbbtdtd6q [NI] OGS (Istituto Nazionale di Oceanografia e di Geofisica Sperimentale) and University of Trieste. (2002). North-East Italy Broadband Network [Data set]. International Federation of Digital Seismograph Networks. 10.7914/SN/NI [OE] ZAMG - Zentralanstalt für Meterologie und Geodynamik. (1987). Austrian Seismic Network [Data set]. International Federation of Digital Seismograph Networks. 10.7914/SN/OE [OX] Istituto Nazionale di Oceanografia e di Geofisica Sperimentale—OGS. (2016). North-East Italy Seismic Network [Data set]. FDSN. 10.7914/SN/OX [RF] University of Trieste. (1993). Friuli Venezia Giulia Accelerometric Network [Data set]. International Federation of Digital Seismograph Networks. 10.7914/SN/RF [SI] ZAMG - Zentralanstalt für Meterologie und Geodynamik. Province Südtirol. https://www.fdsn.org/networks/detail/SI/ [SL] Slovenian Environment Agency. (1990). Seismic Network of the Republic of Slovenia [Data set]. International Federation of Digital Seismograph Networks. 10.7914/SN/SL [ST] Geological Survey-Provincia Autonoma di Trento. (1981). Trentino Seismic Network [Data set]. International Federation of Digital Seismograph Networks. 10.7914/SN/ST [Z3] AlpArray Seismic Network. (2015). AlpArray Seismic Network (AASN) temporary component. AlpArray Working Group. 10.12686/alparray/z3_2015 [ZO] Massa, M., Rizzo, A. L., Lorenzetti, A., Lovati, S., D'Alema, E., Puglia, R., Carannante, S., & Luzi, L. (2021). Rete di monitoraggio multiparametrico del Garda (Nord Italia) - PDnet (Version 1.0) [Data set]. Istituto Nazionale di Geofisica e Vulcanologia (INGV). 10.13127/sd/yhcfomcbo. The following bulletins and databases were consulted: Snidarcig, A., Bernardi, P., Bragato, P.L., Di Bartolomeo, P., Garbin, M., Urban, S., (2016). Bollettino della Rete Sismometrica dell’Italia Nord Orientale (RSINO), [Data set]. Istituto Nazionale di Oceanografia e di Geofisica Sperimentale - OGS. 10.6092/a608d853-755e-4177-aada-992857ccb44e. Snidarcig, A., Bernardi, P., Bragato, P.L., Di Bartolomeo, P., Garbin, M., Urban, S., (2017). Bollettino della Rete Sismometrica dell’Italia Nord Orientale (RSINO), [Data set]. Istituto Nazionale di Oceanografia e di Geofisica Sperimentale - OGS. 10.6092/3ff3c323-d7a4-4183-bea0-1a53814ac8b9 Snidarcig, A., Bernardi, P., Bragato, P.L., Di Bartolomeo, P., Garbin, M., Urban, S., (2018). Bollettino della Rete Sismometrica dell’Italia Nord Orientale (RSINO), [Data set]. Istituto Nazionale di Oceanografia e di Geofisica Sperimentale - OGS. 10.6092/c53f37ce-bcf3-453c-a2cf-1894d48cfbbb Snidarcig, A., Bernardi, P., Bragato, P.L., Di Bartolomeo, P., Garbin, M., Urban, S., (2019). Bollettino della Rete Sismometrica dell’Italia Nord Orientale (RSINO), [Data set]. Istituto Nazionale di Oceanografia e di Geofisica Sperimentale - OGS. 10.6092/58ff169a-2f02-46ae-908a-bdfcacea069c Snidarcig, A., Bernardi, P., Bragato, P.L., Di Bartolomeo, P., Garbin, M., Urban, S., (s2020). Bollettino della Rete Sismometrica dell’Italia Nord Orientale (RSINO), [Data set]. Istituto Nazionale di Oceanografia e di Geofisica Sperimentale - OGS. 10.13120/108b8d94-361a-45f3-8195-fc4e8f73d264 Snidarcig, A., Bernardi, P., Bragato, P.L., Di Bartolomeo, P., Garbin, M., Urban, S., (2021). Bollettino della Rete Sismometrica dell’Italia Nord Orientale (RSINO), [Data set]. Istituto Nazionale di Oceanografia e di Geofisica Sperimentale - OGS. 10.13120/8b252b09-314f-456f-812a-b05268ecd001 Brondi, P., Snidarcig, A., Bernardi, P., Bragato, P.L., Di Bartolomeo, P. (2022). Bollettino della Rete Sismometrica dell’Italia Nord Orientale (RSINO), [Data set]. Istituto Nazionale di Oceanografia e di Geofisica Sperimentale - OGS. 10.13120/w1vp-b578 CRS-OGS staff (2024, October). Real Time Seismology of the OGS Seismological Research Centre website. https://rts.crs.inogs.it ISIDe Working Group (2007). Italian Seismological Instrumental and Parametric Database (ISIDe) (Version 1). Istituto Nazionale di Geofisica e Vulcanologia (INGV). 10.13127/ISIDE
