# Supplementary material for: Spatiotemporal variations in b-value suggest an evolving mechanical state of the crust in the southeastern Alps
Source: Sci Rep. 2026 May 20;16:23047. doi: 10.1038/s41598-026-51916-x (PMC13392025; doi:10.1038/s41598-026-51916-x)
Supplement: Supplementary file 1 — Supplementary Information. [file 41598_2026_51916_MOESM1_ESM.pdf]

Supplemental material for

Spatiotemporal variations in b-value suggest an evolving mechanical state of the crust in the southeastern Alps

Picozzi M.*, Spallarossa D., Bindi D.

*Corresponding author. Email: mpicozzi@ogs.it

This file includes: 16 Figures (from Fig. S1 to Fig. S16) and 4 Tables (from Table S1 and Table S4).

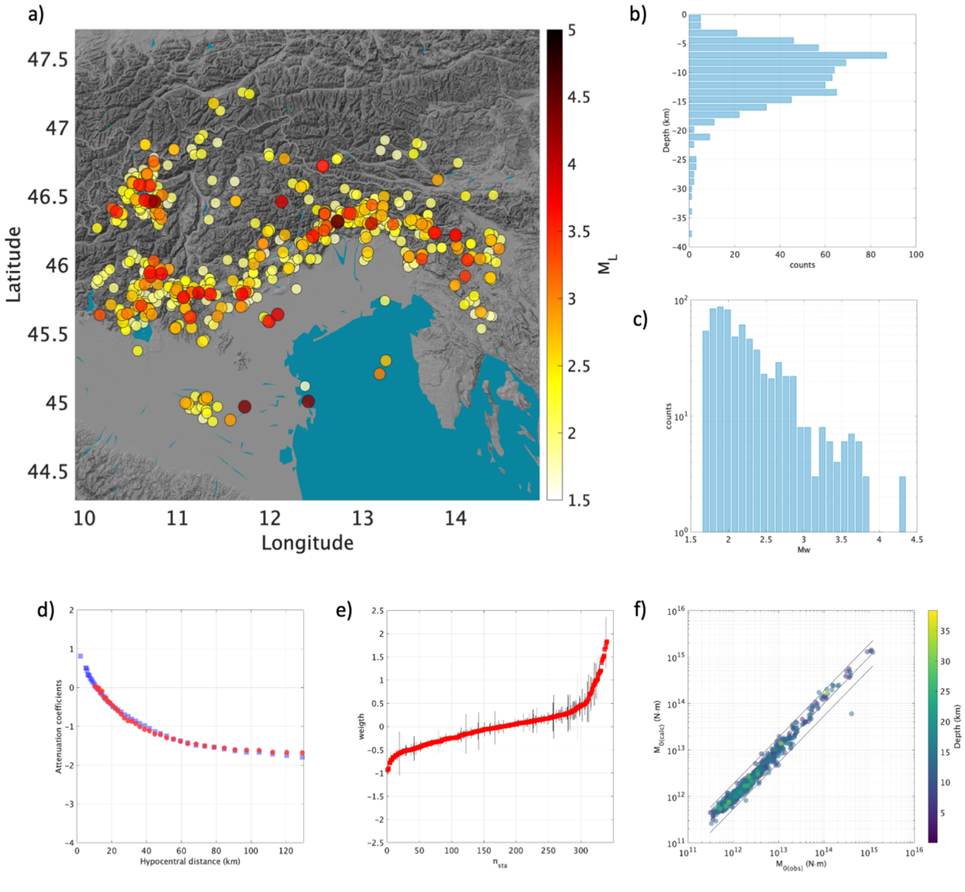

Figure S1. a) Distribution of events colored per ML from Cataldi et al. (2025) used to calibrate the attenuation and path correction terms for the estimation of M_0 in the area of study. b) Distribution of hypocentral depths. c) Frequency magnitude distribution of Mw. e) Comparison between the attenuation curve for southeastern Alps (blue) and central Italy (red), (see Methods). e) Station correction coefficients (red) and their standard deviations (black vertical bars). f) Comparison of M_0 from Cataldi et al. (2025) and the calibrated model for estimating it from S-wave peak displacement (see Methods).

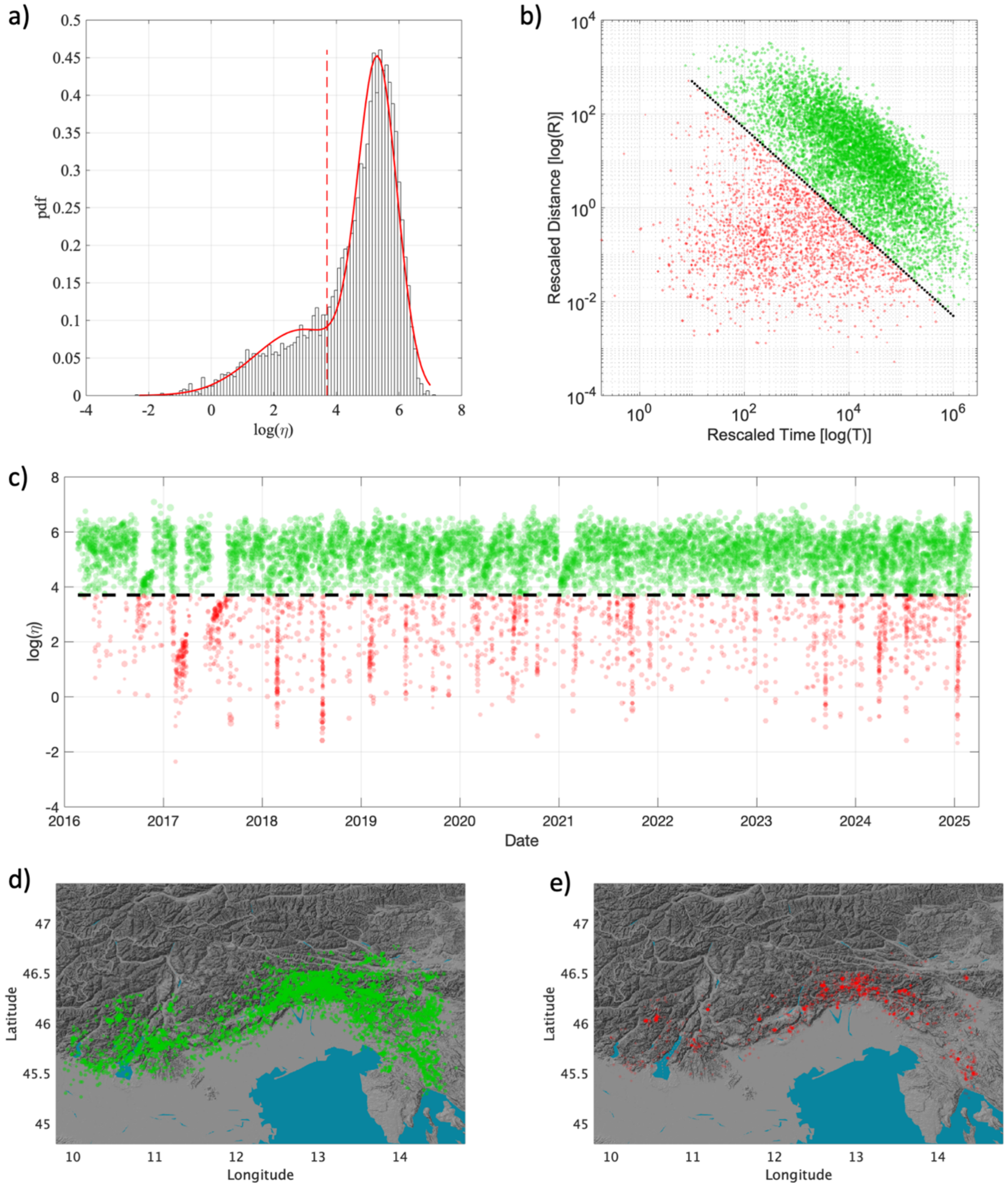

Figure S2. Clustering analysis for identification of background and clustered seismicity. a) Histogram of the nearest-neighbor distance h, which is modeled by the sum of two log-Gaussian function (red line). Threshold h value considered for discriminating the two populations (red dashed line). b) Rescaled time versus Rescaled Distance for background (green) and clustered seismicity (red). Threshold h value considered for discriminating the two populations (black dotted line). c) the same as b) but temporal evolution of h. d) Distribution of events belonging to background. (e) The same as (d), but for the clustered seismicity.

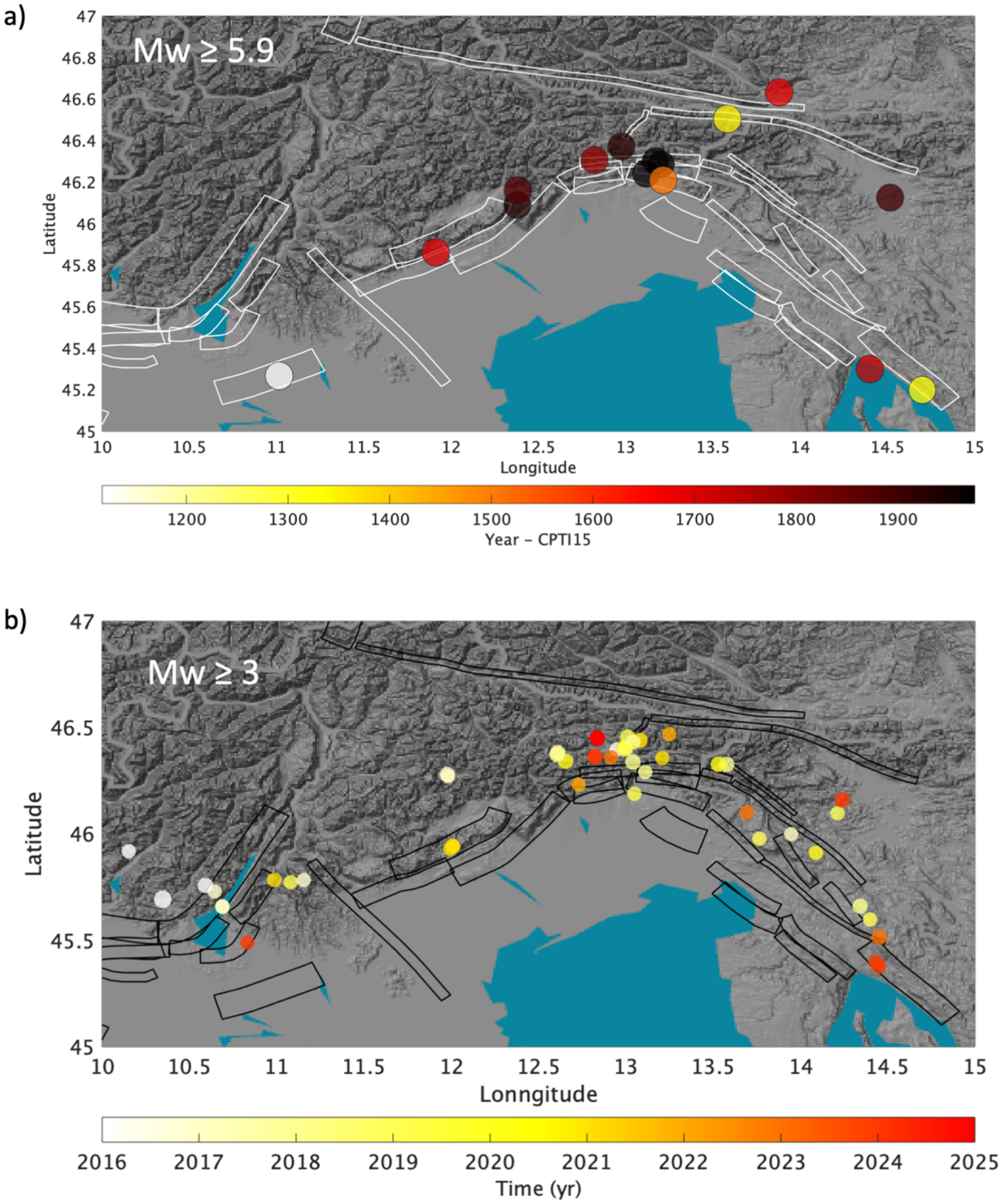

Figure S3. a) Distribution of historical earthquakes with Mw >= 5.9 (Rovida et al., 2022) colored per occurrence year. Seismogenic sources from DISS (https://diss.ingv.it). b) the same as a) but for earthquakes with Mw >= 3 recorded in the period 2016-2025.

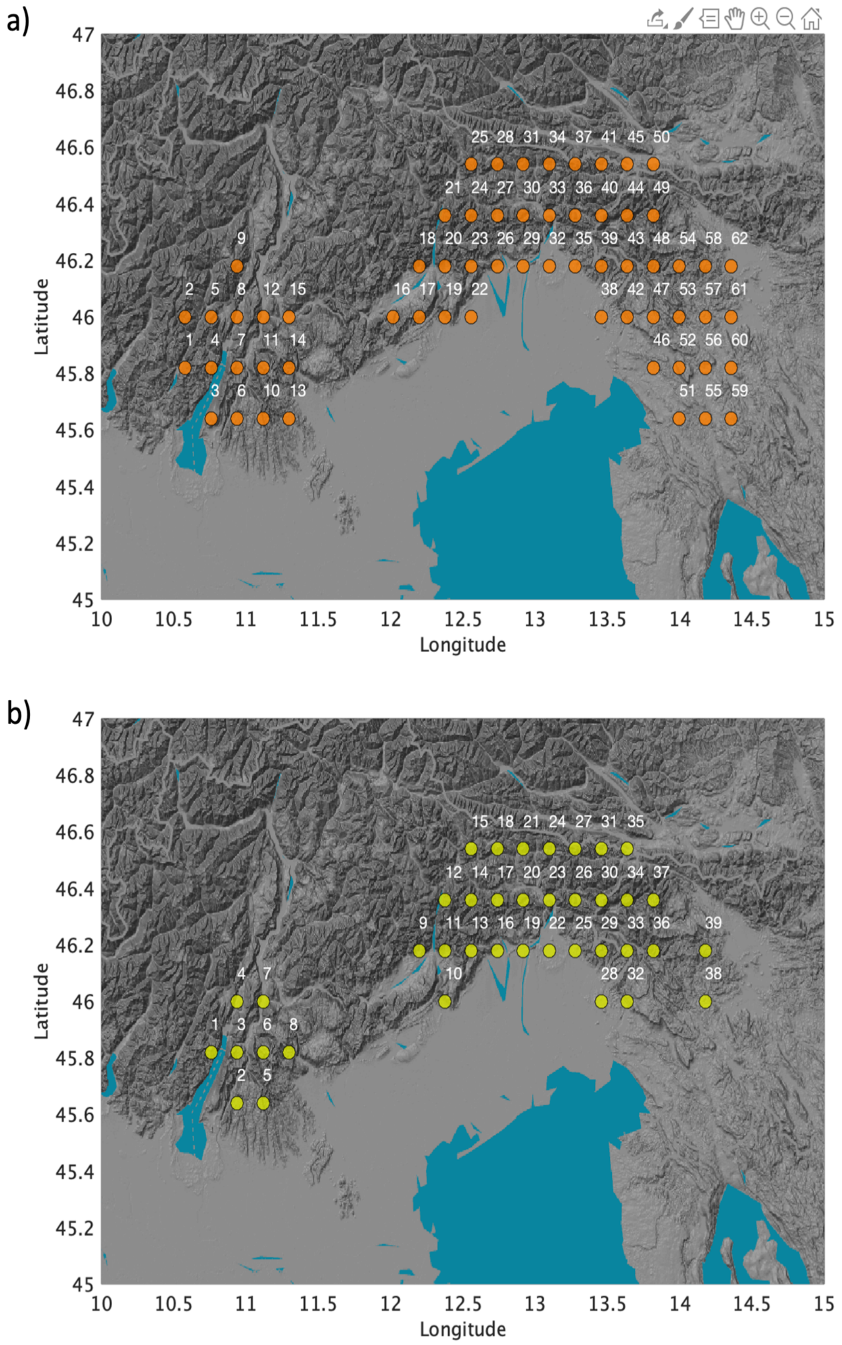

Figure S4. a) Grid nodes distribution for which b-value is estimated (the node number is the reference to the figures with the frequency magnitude distribution and b-value estimation that are uploaded separately as Supplemental Material). b) The same as a), but for node for which it is estimated Db.

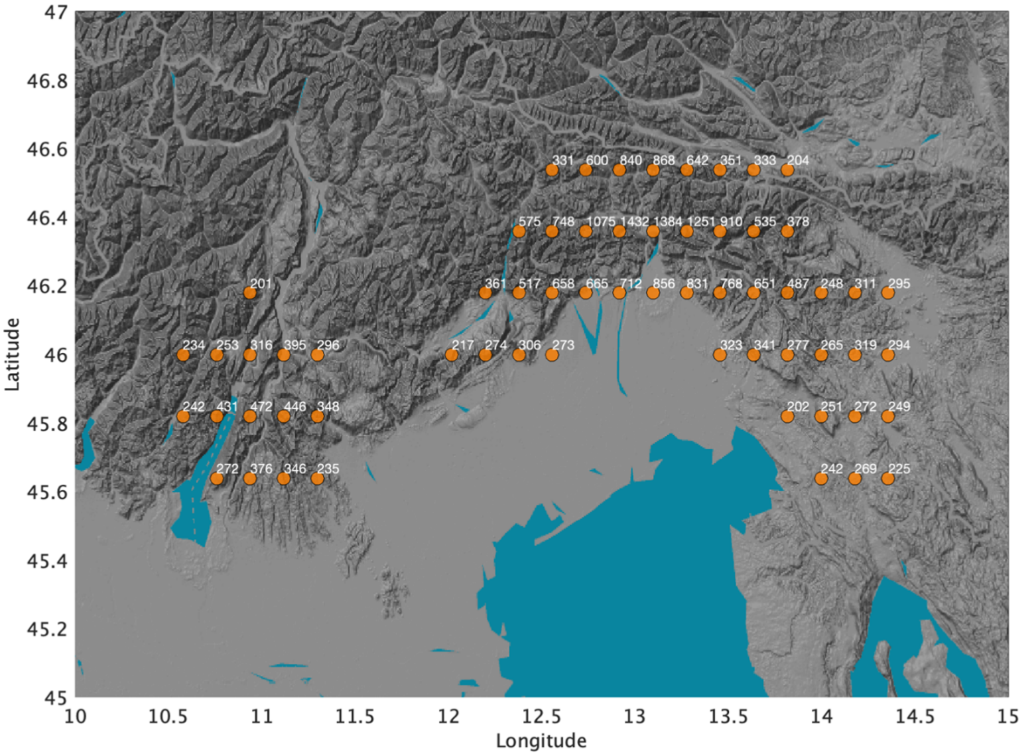

Figure S5. Number of events per node.

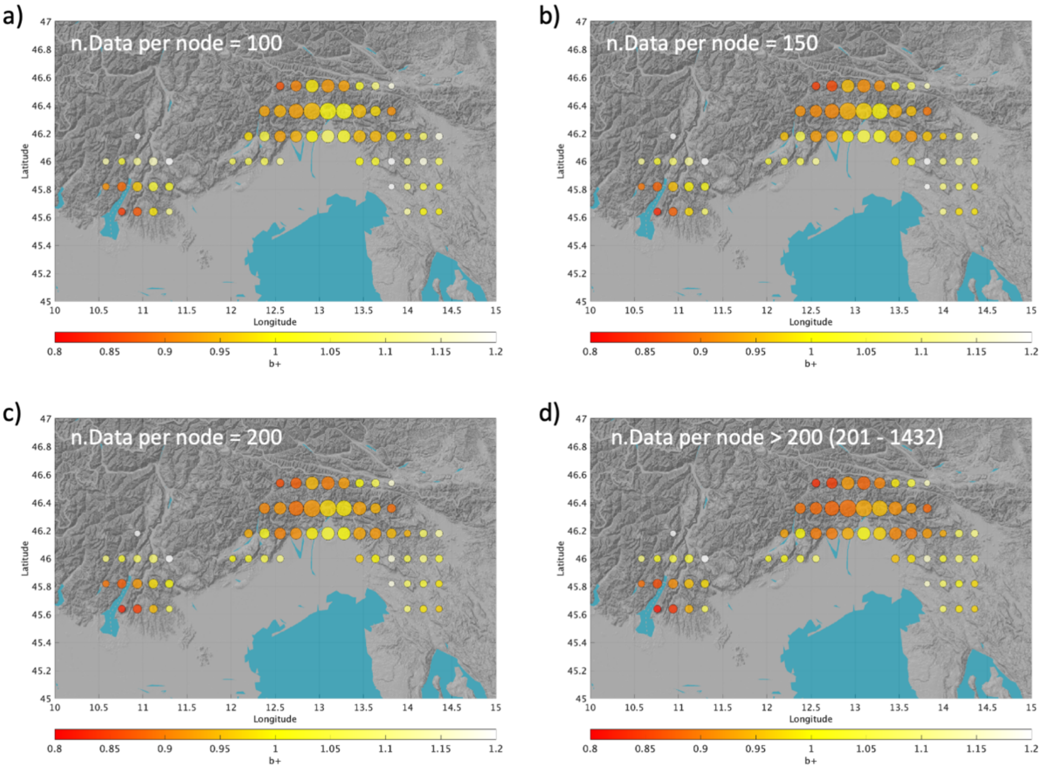

Figure S6. Maps showing the b-value estimates for background seismicity over a regular grid using different number of data associated to the nodes: a) 100 event per node, b) 150, c) 200, and d) variable with the condition >200 (i.e., from 201 to 1432).

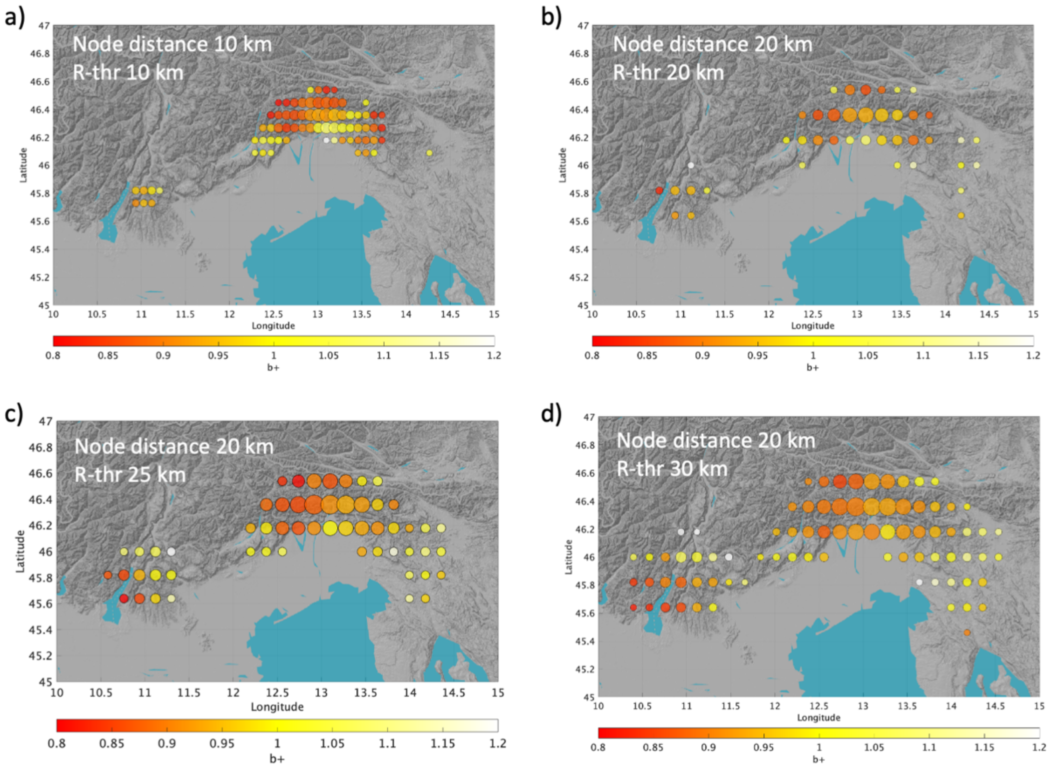

Figure S7. Maps showing the b-value estimates for background seismicity over a regular grid using different parameters for the grid node spacing and radius for associating events to a node, R-thr: a) grid node spacing 10 km, R-thr 10 km, b) grid node spacing 20 km, R-thr 20 km, c) grid node spacing 20 km, R-thr 25 km (which is the one selected as Fig. 1d), and d) grid node spacing 20 km, R-thr 30 km.

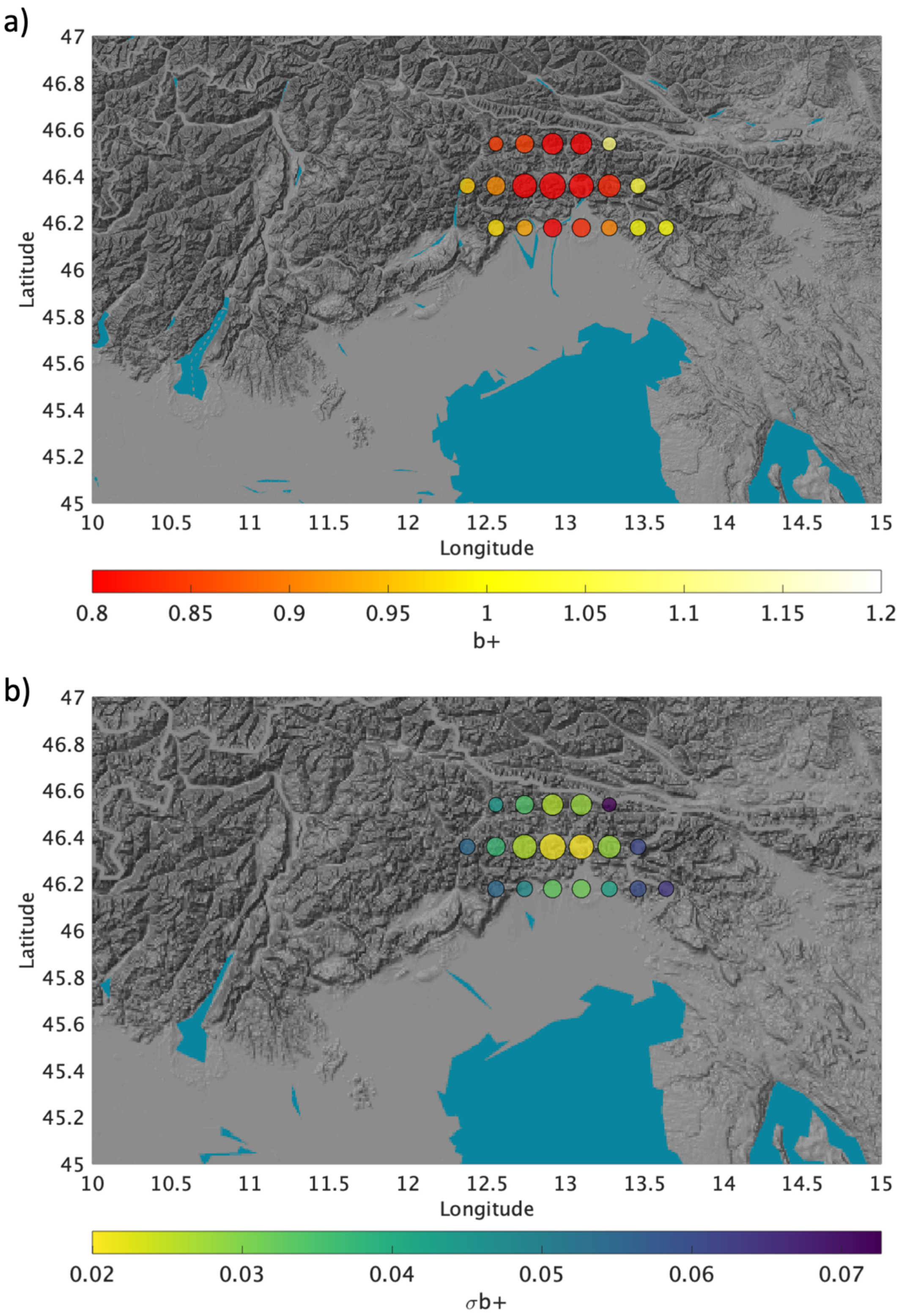

Figure S8. a) Map showing the mean b-values from bootstrap for clustered seismicity over a regular grid. The dimension of dots is related to the amount of data for grid nodes. b) The same as a), but for b-value uncertainty.

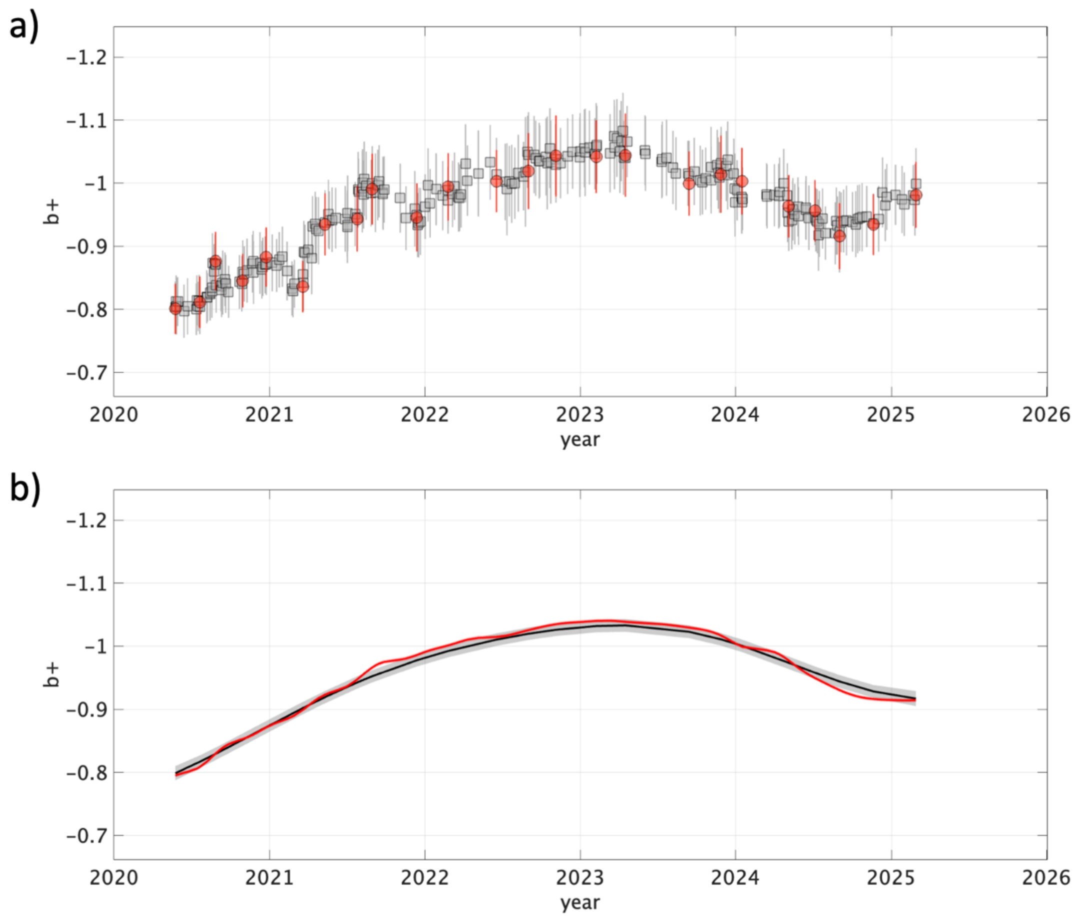

Figure S9. a) comparison between b-value time series using a window shift equal to 10 events (red dots) and 1 event (gray squares). b) comparison between b-value trends for the two window shift parameters using the same color of panel a).

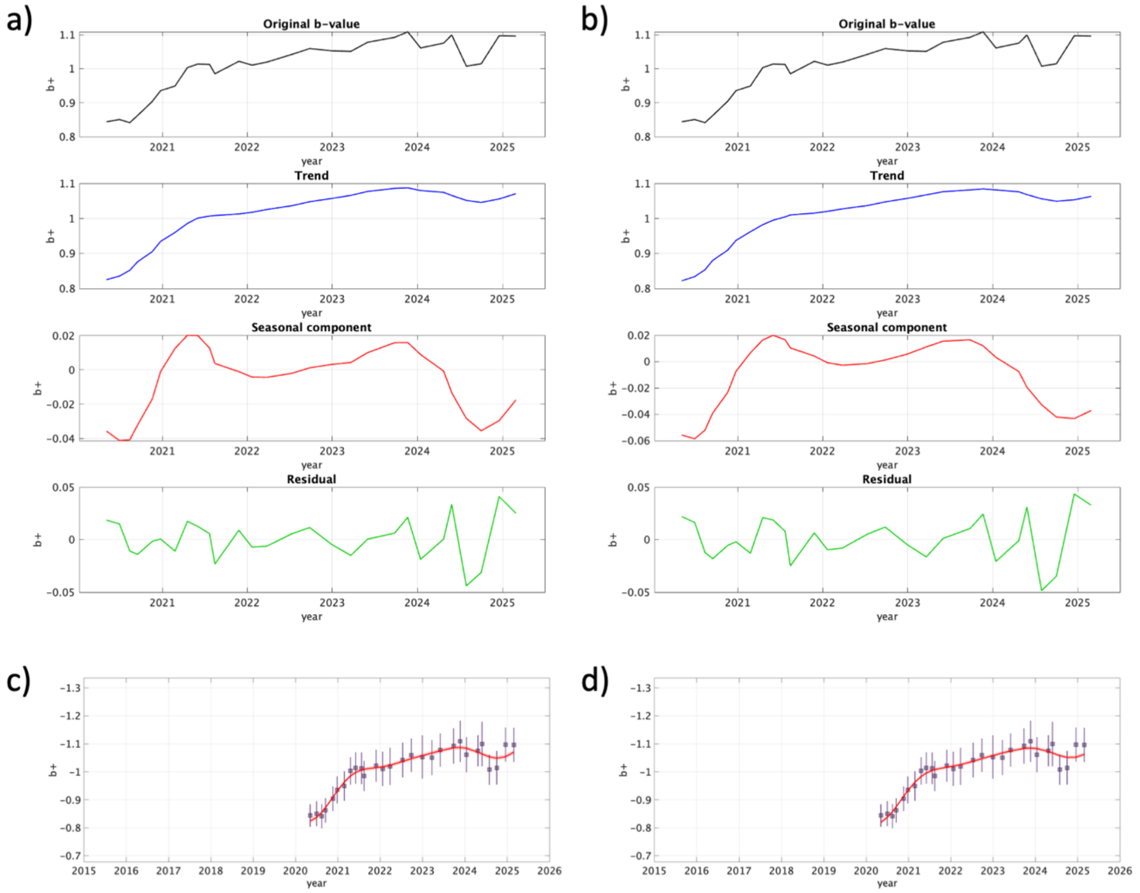

Figure S10. Examples of SSA Analysis on b-value time-series of two grid nodes. The left panels a) and c) show the results for L equal to 10, while panels b) and d) for L equal to 20.

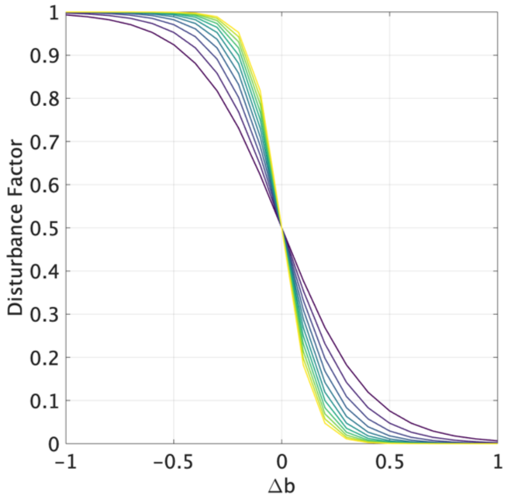

Figure S11. Logistic function relating Δb to the Disturbance Factor for k varying between 5 (yellow) and 15 (blue).

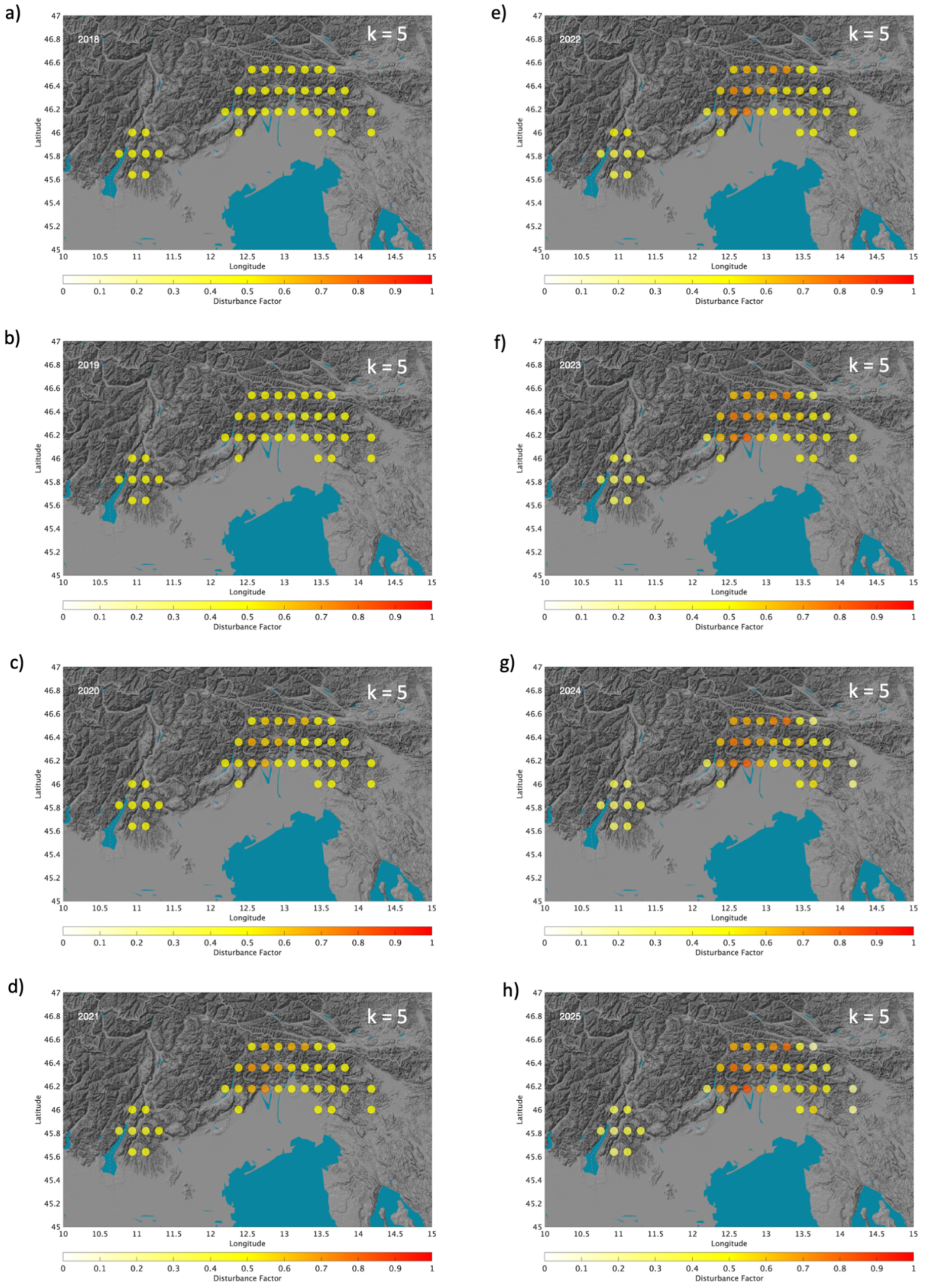

Figure S12. Spatio-temporal evolution of the Disturbance factor. From a) to h), maps showing D over a regular grid using k equal to 5.

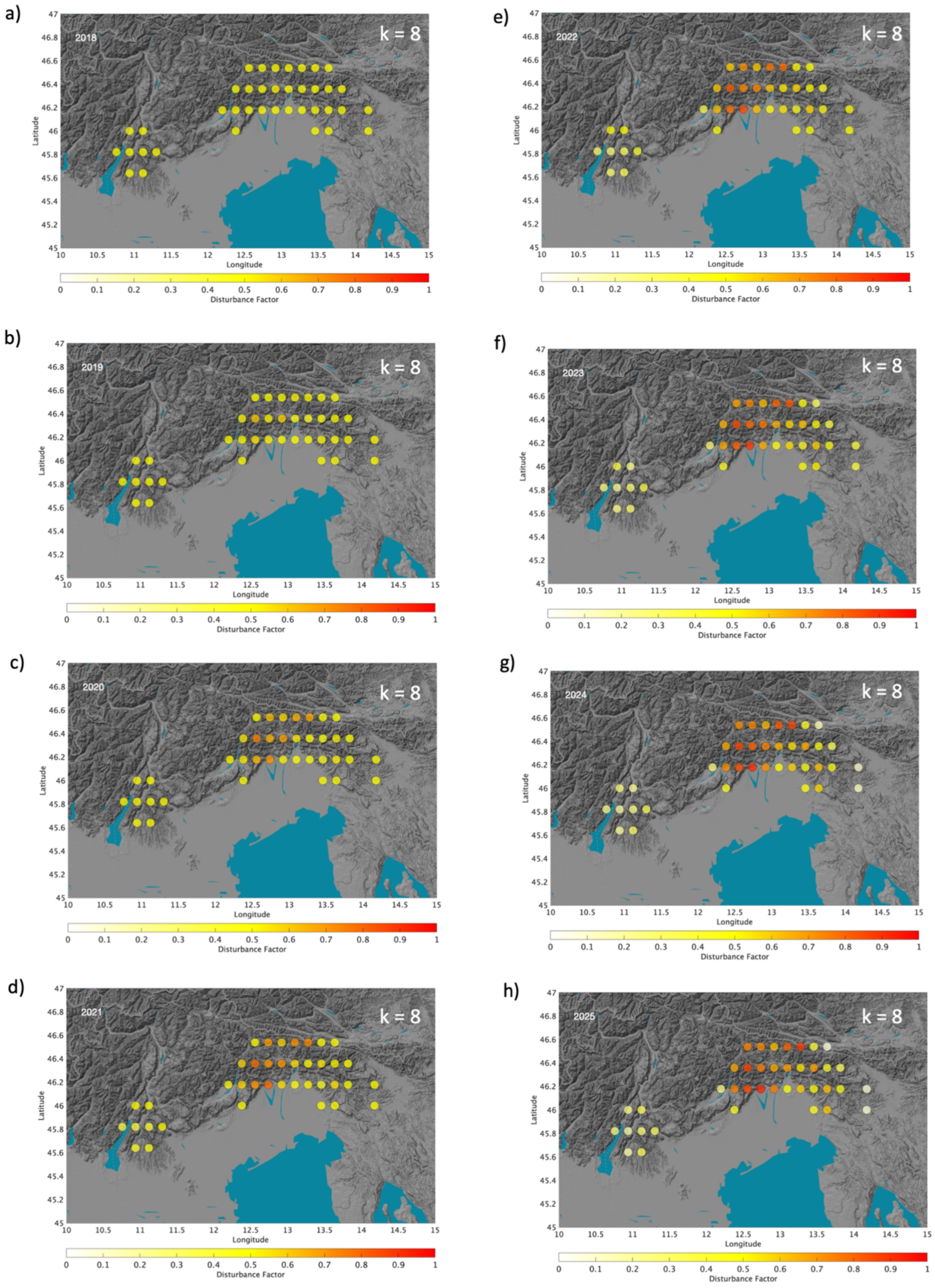

Figure S13. Spatio-temporal evolution of the Disturbance factor. From a) to h), maps showing D over a regular grid using k equal to 8.

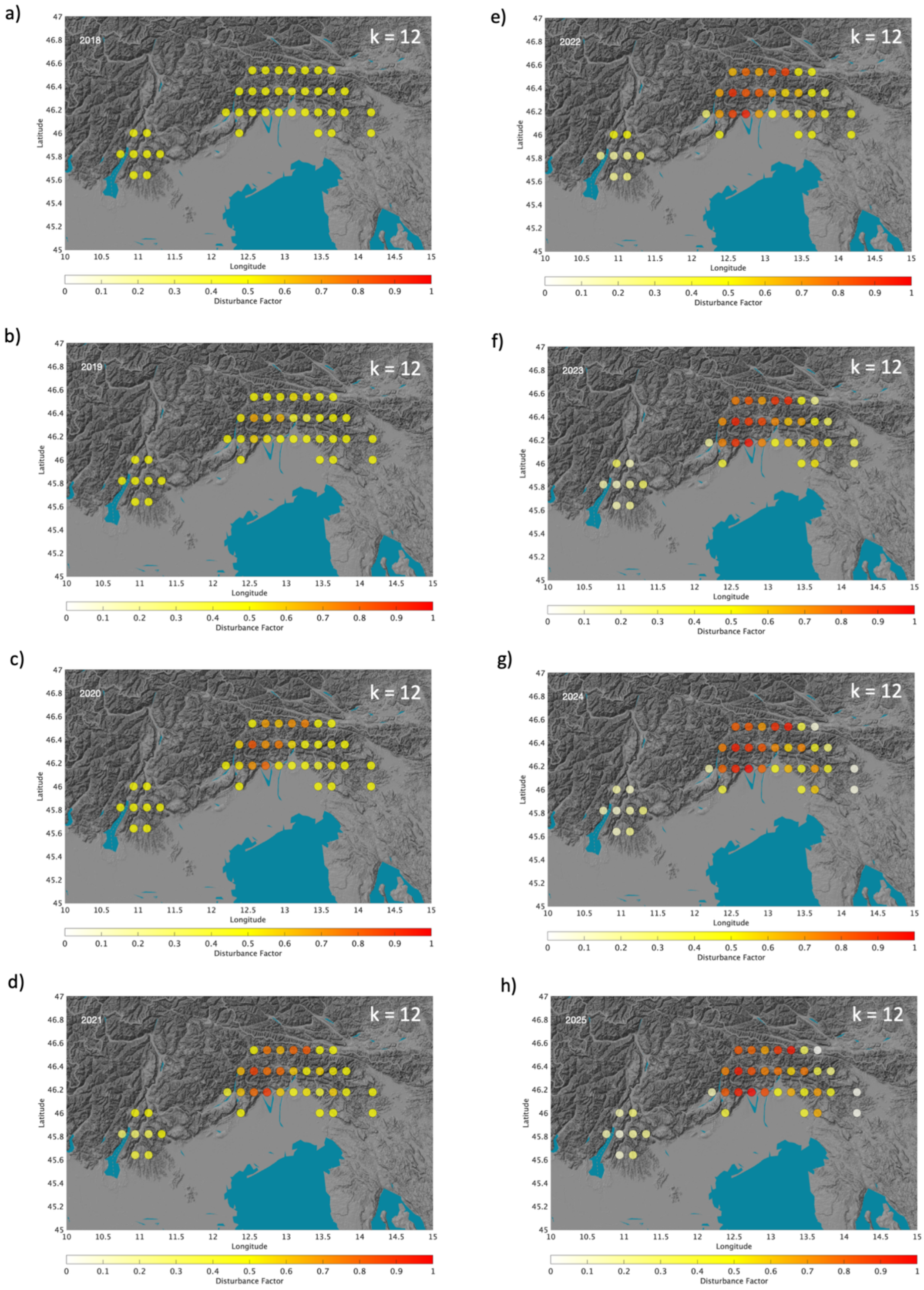

Figure S14. Spatio-temporal evolution of the Disturbance factor. From a) to h), maps showing D over a regular grid using k equal to 12.

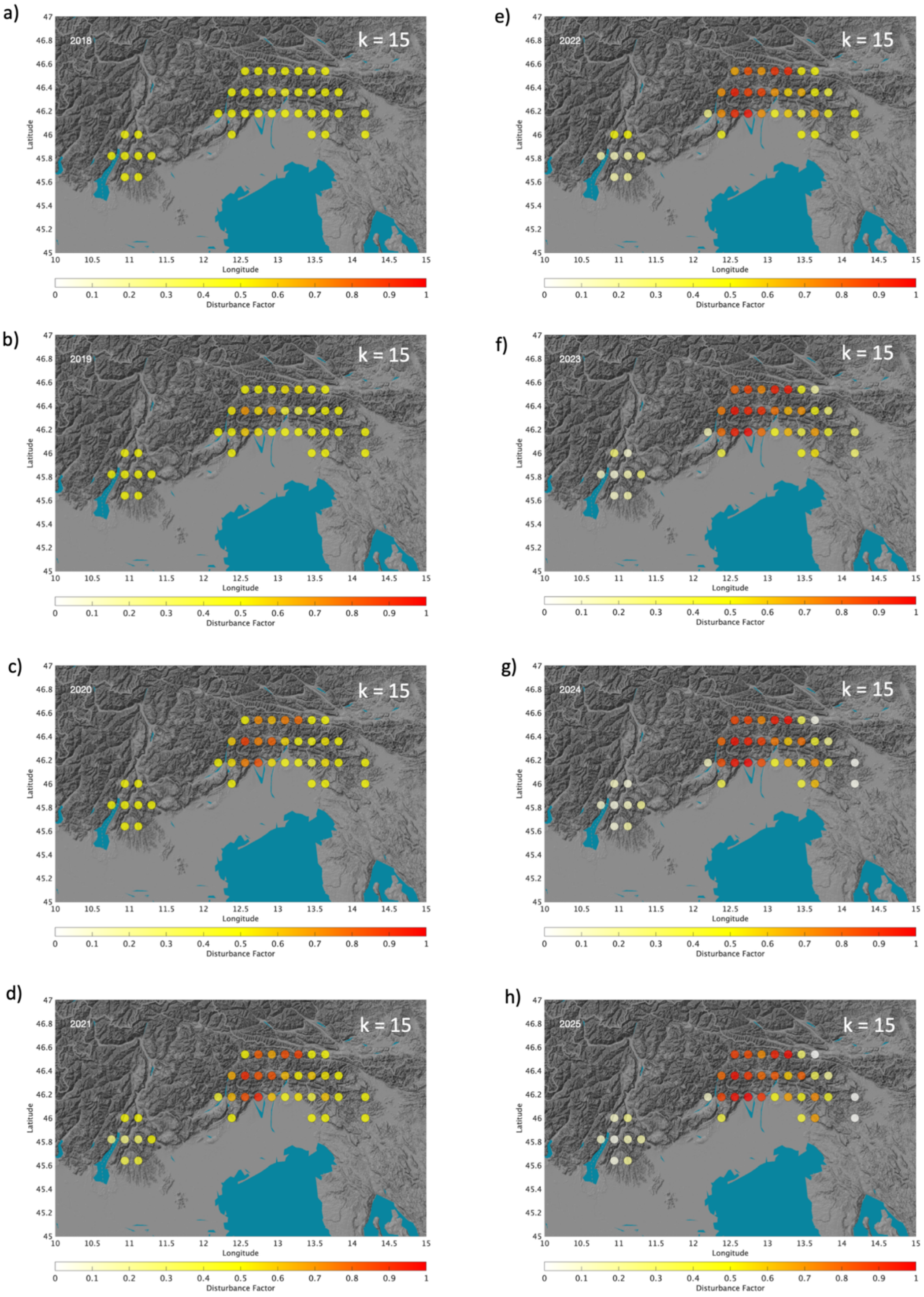

Figure S15. Spatio-temporal evolution of the Disturbance factor. From a) to h), maps showing D over a regular grid using k equal to 15.

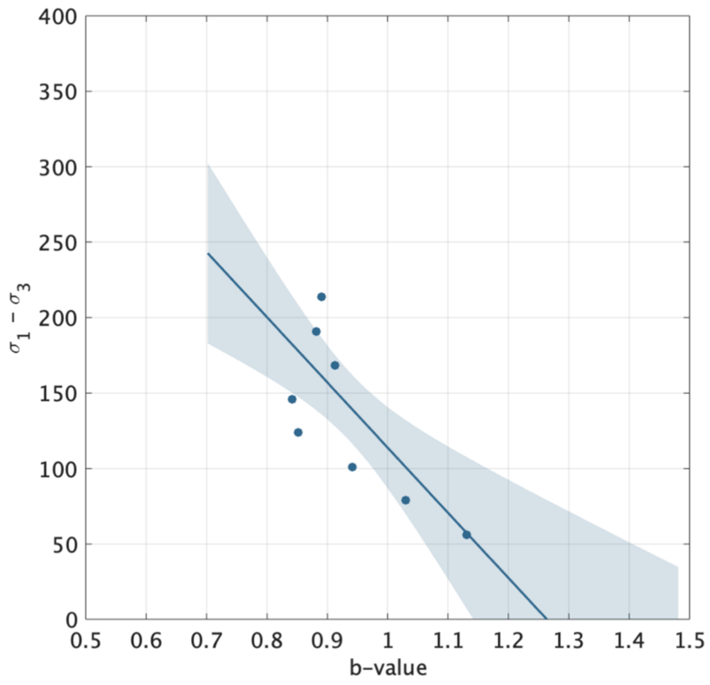

Figure S16. Calibration of the b-value to differential stress (σ_D) relation (blue line) +/- one standard deviation (light blue area) for Italian data (blue dots) following Scholz (2015).

Table S1. The station corrections for PD_S (S in Eq.1) and uncertainties. The Station code format is: 2 characters as network code, 3-4 characters station code, 2 characters sensor typology.

Station code	Station weight for PD_S	uncertainty
SL.LJU.HH	0,28346	0,01828
SL.GBAS.HH	-0,033953	0,018818
SL.CRNS.HH	0,3168	0,019423
SL.MOZS.HH	0,078606	0,019854
SL.JAVS.HH	0,085398	0,016555
SL.VOJS.HH	0,20527	0,017712
SL.VISS.HH	0,07072	0,015056
SL.GORS.HH	0,07924	0,016248
OE.OBKA.HH	-0,49248	0,037207
SL.CADS.HH	0,16996	0,017997
SL.KNDS.HH	0,076917	0,01465
SL.SKDS.HH	0,16781	0,022437
SL.GBRS.HH	0,44345	0,021032
SL.ROBS.HH	-0,21382	0,016221
OX.PRED.HH	-0,66805	0,02509
SL.CRES.HH	-0,0352	0,016614
OE.SOKA.HH	-0,52628	0,049917
SL.PERS.HH	0,21793	0,022377
OE.MYKA.HH	-0,61955	0,025444
SL.BOJS.HH	-0,0022115	0,0173
OX.ACOM.HH	-0,078051	0,02106
SL.GCIS.HH	0,058649	0,021155
SL.GROS.HH	0,11521	0,027435
Z3.A250A.HH	-0,26713	0,12132
IV.BRMO.HH	-0,40883	0,026558
SI.MOSI.HH	-0,052135	0,021815
CH.FUORN.HH	-0,52142	0,019733
IV.MABI.HH	-0,46276	0,020508
CH.BERNI.HH	-0,55407	0,022301
ST.GAGG.HH	-0,24144	0,016794
IV.APPI.EH	-0,13681	0,020448
CH.SARD.HG	0,15384	0,039065
SI.LUSI.HH	-0,0025411	0,015552
SI.KOSI.HH	-0,36799	0,023971
SI.ABSI.HH	-0,11694	0,020105
IV.BAG8.HN	0,13299	0,078099
OE.FETA.HH	-0,6257	0,027458

CH.DAVOX.HH	-0,39839	0,033544
CH.SBGN.HG	0,057772	0,048863
IV.MAGA.HH	0,075387	0,018922
ST.DOSS.HH	-0,14574	0,015547
Z3.A289A.HH	-0,20759	0,025463
OX.GARG.EH	-0,34206	0,028394
IV.ZONE.HH	-0,053037	0,024071
OX.BALD.HH	0,085541	0,024403
SI.ROSI.HH	-0,25942	0,019593
IV.VOBA.HN	0,11462	0,054971
CH.SVAM.HG	0,028531	0,0595
IV.SALO.HH	-0,18003	0,024212
IV.CTI.HH	-0,01751	0,01538
CH.SCHK.HG	0,14222	0,048743
IV.ROVR.HH	-0,27946	0,025044
MN.TUE.HH	-0,44469	0,036926
OX.MARN.HH	-0,086636	0,019273
Z3.A291A.HH	-0,36172	0,021175
OE.SQTA.HH	-0,33807	0,029169
OX.FAU.SH	-0,4328	0,022687
OE.MOTA.HH	-0,46358	0,058
OX.CGRP.HH	-0,24156	0,024147
CH.STRW.HG	0,043089	0,048099
OX.AGOR.HH	-0,32934	0,019669
Z3.A062A.HH	0,21176	0,46999
OE.RETA.HH	-0,66367	0,057401
OX.AFL.SH	-0,26516	0,025035
OE.ABTA.HH	-0,51748	0,017782
OX.CSM.SH	-0,30519	0,040857
IV.FVI.HH	-0,29392	0,021349
OX.CIMO.HH	-0,70003	0,016131
OX.CSO.SH	-0,50265	0,024353
OX.CLUD.HH	-0,58581	0,017885
SI.RISI.HH	0,019209	0,03448
IV.STAL.HH	-0,24588	0,016542
OX.ZOU2.HH	-0,35115	0,022409
OX.FUSE.HH	-0,40582	0,016989
OX.MLN.HH	-0,57938	0,021151
OX.PLRO.HN	-0,056575	0,049014
OX.MPRI.HH	-0,36694	0,018699
OX.BOO.HN	0,096244	0,09615
RF.GEPF.HH	-0,35987	0,018302

OX.VARN.HH	-0,12318	0,015662
IV.PTCC.HH	-0,47236	0,023204
OE.KBA.HH	-0,61352	0,024262
OE.WTTA.HH	-0,48438	0,032502
OE.WATA.HH	-0,55559	0,045614
OX.DRE.HH	-0,025318	0,014707
SL.DOBS.HH	0,3406	0,020861
OX.SABO.HH	-0,10638	0,015503
OX.COLI.SH	0,14637	0,039855
IV.BRES.EH	-0,24071	0,02157
SI.BOSI.HH	0,24792	0,036685
CH.SCUC.HG	0,1506	0,060607
CH.STSP.HG	0,095423	0,058914
CH.SZER.HG	0,12641	0,11437
OX.TOPP.02HN	-0,088489	0,047259
OX.BUA.SH	-0,24574	0,035463
RF.MOGG.HN	-0,25534	0,025201
OX.BAD.HN	-0,23686	0,057268
IV.ASOL.HN	0,037135	0,022922
Z3.A021A.00HH	0,22446	0,14214
Z3.A020A.00HH	-0,27927	0,014011
SL.KOGS.HH	0,27062	0,014075
OX.LSR.SH	-0,05809	0,035217
OX.CAE.HH	-0,32033	0,019361
OX.MTLO.SH	0,20617	0,045406
IV.ZEN8.HN	-0,26973	0,062064
OX.GAVI.EH	0,13581	0,044234
OX.TRES.EH	-0,053506	0,054881
IV.TREG.HN	0,17053	0,029462
IV.CNCS.HN	0,25414	0,11601
Z3.A313A.HH	0,086755	0,092315
IV.OPPE.HH	0,16163	0,064695
IV.CAPR.HN	0,068167	0,083478
IV.ZOVE.HN	-0,10689	0,033364
IV.SANR.HN	0,22774	0,080858
IV.MDI.HH	-0,26007	0,024129
IV.TEOL.HH	0,20088	0,026044
IV.CRND.HN	-0,15185	0,034629
OX.MIRB.02HN	0,3108	0,15637
IV.CAVE.HH	0,20632	0,077241
IV.MERA.HN	0,2373	0,05503
CH.SCEL.HG	0,077881	0,049951

NI.VINO.HH	-0,3851	0,015679
IV.BORM.HN	-0,011812	0,1429
CH.SDAK.HG	0,4231	0,090565
CH.SCUG.HG	-0,011445	0,075893
OE.DAVA.HH	-0,44757	0,037528
CH.PLONS.HH	-0,095244	0,40565
CH.SBUB.HG	-0,71875	0,01339
CH.LIENZ.HH	-0,51247	0,070353
CH.SBUH.HG	1,1485	0,18713
RF.GORI.HN	0,21215	0,029652
OX.GAZZ.SH	0,067273	0,063433
IV.FERS.HN	0,17282	0,13272
IV.CMPO.HN	-0,048001	0,1036
GU.GSCL.HH	-0,41664	0,081387
IV.FNVD.HH	-0,42887	0,10953
IV.LMD.HH	-0,49786	0,12504
IV.MPPT.EH	-0,45499	0,0143
IV.BDI.HH	-0,77709	0,2382
IV.LEOD.HN	0,16549	0,097408
IV.FRE8.HN	-0,28486	0,036264
RF.MORT.HH	-0,64673	0,027799
IV.NDIM.HN	0,3568	0,15233
NI.POLC.HH	-0,038424	0,023448
Z3.A302A.HH	1,4665	0,056023
RF.CARC.HN	0,90414	0,13234
Z3.A300A.HH	1,498	0,061612
Z3.A253A.HH	-0,45282	0,047668
Z3.A301A.HH	1,5298	0,097083
CR.BRJN.HH	0,13418	0,27877
OX.GRDM.EH	0,18512	0,037871
IV.SERM.EH	0,30904	0,38577
OX.TEOL.SH	0,28644	0,13595
IV.VENL.HN	0,33739	0,20134
IV.RAVA.EH	0,39289	0,12051
IV.MODE.HN	0,18026	0,070047
OX.ADRI.SH	0,25964	0,039024
IV.ZCCA.HH	-0,48173	0,11856
IV.PRMA.HH	-0,3398	0,068382
Z3.A285A.HH	-0,034728	0,06887
MN.TRI.HH	-0,24867	0,026603
IV.NDIM.HH	0,23985	0,053364
OX.PLRO.HH	-0,014521	0,016603

IV.FIU.EH	0,14601	0,04593
Z3.A312A.HH	0,25249	0,17676
IV.BRIS.HH	-0,37805	0,095947
IV.SBPO.HN	0,4413	0,16643
IV.MTRZ.HH	-0,15576	0,099803
IV.SEI.HH	-0,32129	0,23815
GU.POPM.HH	-0,51111	0,062033
MN.VLC.HH	-0,78244	0,013912
IV.MOCL.EH	-0,72329	0,013003
OX.APGO.EH	-0,28161	0,024266
OX.BAD.HH	-0,33699	0,017824
IV.ORZI.HH	0,1777	0,13143
IV.BOTM.HN	-0,35415	0,084331
OX.QUIN.EH	0,096081	0,093176
OX.MTLO.HH	0,16861	0,025815
CH.MUGIO.HH	-0,46324	0,1457
IV.BOB.HH	-0,15919	0,42922
RF.MONF.HH	-0,25511	0,071161
IV.MILN.HH	0,81732	0,23442
NI.PURA.HH	-0,57086	0,025168
NI.DST2.HH	-0,14933	0,037557
OX.BOO.HH	-0,23029	0,017723
OX.CSM.HH	-0,33297	0,017553
OE.LESA.HH	-0,50992	0,03282
IV.MBAL.EH	-0,039509	0,023727
ST.RONC.EH	0,032103	0,026363
ST.CARE.EH	-0,50662	0,020487
ST.ZIAN.EH	-0,23473	0,021691
ST.PANI.EH	-0,33344	0,018424
ST.VARA.EH	0,038089	0,019986
ST.OZOL.EH	-0,0013412	0,020954
CH.SIZS.HG	0,37056	0,20119
Z3.A284A.HH	0,57113	0,012964
CH.PANIX.HH	-0,47499	0,26039
OX.IESO.SH	0,14581	0,055931
RF.PALM.HN	0,10335	0,060697
CH.SMFL.HG	1,8082	0,55301
IV.VARE.HH	-0,22532	0,11373
SL.VNDS.HH	0,17147	0,015676
IV.CADC.HN	0,43842	0,2227
NI.PALA.HH	-0,10749	0,018111
RF.PAUL.HN	-0,049523	0,045145

SL.CEY.HH	0,076522	0,027289
CH.SWAS.HG	1,205	0,013817
SL.ZAVS.HH	0,47525	0,019988
Z3.A020B.00HH	-0,11717	0,012323
OX.STIN.HH	-0,10953	0,052468
OX.CSO.EH	-0,3818	0,032834
OX.BUA.EH	-0,25544	0,032354
CH.SMELS.HG	1,1727	0,14717
OX.ADRI.EH	0,45522	0,078786
OX.COLI.EH	0,03734	0,02379
Z3.A303A.HH	1,7146	0,012174
OX.LSR.EH	-0,16095	0,021767
OX.CEKR.EH	-0,40973	0,0266
OX.GAZZ.EH	-0,097879	0,18363
CH.SUSI.HG	0,80339	0,015904
CH.SLOP.HG	1,3681	0,012256
OE.BIOA.HH	-0,38904	0,042558
IV.MNTV.HN	0,12186	0,15282
OX.IESO.EH	0,15322	0,098641
IV.NEVI.HN	0,28846	0,40442
IV.BOZZ.HN	1,2003	0,013296
IV.ZOVE.EH	-0,17763	0,042602
CH.VDR.HH	-0,55005	0,032345
CH.SBUA2.HG	0,10401	0,014525
IV.FREG.EH	-0,094727	0,042562
OX.ZOU.EH	-0,42345	0,032105
IT.VNZ.HN	0,026355	0,048829
IV.BAG8.EH	-0,012912	0,0278
IV.VMAN.HH	-0,058624	0,15052
IV.BOZZ.EH	0,2528	0,17803
IV.SGAL.HN	0,04147	0,014937
IV.TREG.EH	0,018639	0,033347
IV.SGAL.EH	-0,25181	0,032066
RF.CESC.HN	-0,0086057	0,021046
RF.STOL.HN	0,26013	0,03311
IV.ZEN8.EH	-0,49032	0,031853
IV.VOBA.EH	0,0084172	0,040609
IV.FERS.EH	0,2435	0,11501
RF.PRAD.HN	-0,32595	0,033905
RF.MASA.HN	0,0070193	0,031933
RF.GESC.HN	0,21246	0,056005
RF.GEDE.HN	0,29532	0,043425

IV.CNCS.EH	-0,054659	0,036321
IV.MNTV.EH	0,34501	0,20059
IV.VISG.HH	-0,89395	0,075716
GU.EQUI.HH	-0,54302	0,024515
IV.CADC.EH	0,33052	0,13746
RF.POR.HN	0,50524	0,13206
IT.ZPP.HN	0,13131	0,10246
IV.VENL.EH	0,70676	0,37422
ZO.PDN6.EH	-0,19075	0,10076
ZO.PDN3.EH	-0,11503	0,031478
ZO.PDN2.EH	-0,089927	0,058518
ZO.PDN1.EH	0,20689	0,30732
IV.PCN.EH	-0,029454	0,35857
IV.ASOL.EH	0,011277	0,047375
CH.VMV.HH	-0,39337	0,050106
CH.SWERG.HG	1,4279	0,012914
IV.MBAL.HH	-0,08039	0,046214
ZO.PDN8.EH	0,018468	0,072076
ZO.PDN7.EH	-0,13938	0,041196
OX.MGBU.HH	-0,24365	0,042575
GU.GRAM.HH	-0,61268	0,11383
ZO.PDN4.EH	-0,0078293	0,074133
IV.SANR.EH	0,11561	0,070865
Y1.T1757.EH	0,15833	0,014428
Y1.T1756.EH	-0,19877	0,014441
IV.VMGN.HH	-0,9279	0,10976
IV.SFI.EH	-0,31339	0,015064
IT.VRNA.HN	1,0087	0,15216
OX.AFL.EH	0,4071	0,7235
OX.TLI.EH	0,020322	0,048774
4P.IT07A.HH	-0,046651	0,14425
RF.GEPF.HN	-0,14049	0,12036
4P.IT09A.HH	0,35379	0,19285
4P.IT08A.HH	0,52885	0,026338
IT.MLC.HN	0,082667	0,20111
IT.CHF.HG	-0,18047	0,16174
OE.ARSA.HH	-0,54123	0,014868
IT.DRN.HN	-0,029911	0,13481
IT.CMO.HN	-0,17644	0,079535
IT.REA.HN	-0,067704	0,013254
IT.SPI.HN	-0,17113	0,013398
IT.PSR.HN	0,53853	0,43853

IT.FIC.HN	-0,66236	0,026134
IT.GAI.HN	0,25212	0,10275
IT.SNZ1.HG	0,67704	0,023089
IT.FAG.HN	0,9848	0,1067
IT.LTSN.HN	0,96087	0,016601
IT.CVF.HN	-0,1624	0,090342
OX.STIN.HN	1,6677	0,016101
IT.PAS.HN	0,69526	0,015396
IT.PON.HN	0,48443	0,23534
IT.TLM2.HG	0,046192	0,0395
IT.SDF.HN	-0,23964	0,014401
IT.RVST.HN	0,46205	0,35108
IT.OVAR.HN	0,60964	0,51384
IT.TGG.HG	-0,14508	0,04224
IT.CST.HG	1,8238	0,011403
IT.MNS.HG	0,00058859	0,13837
IT.SUZR.HN	0,47578	0,1113
IT.NVL.HG	0,52891	0,014491
IT.AVS.HN	-0,32231	0,084375
IV.FELT.EH	-0,15263	0,037149
IT.MRN.HG	0,12522	0,063216
IT.CRP.HN	0,8806	0,014903
ZO.PDN10.EH	-0,059717	0,014037
ZO.PDN13.EH	-0,50018	0,058399
IT.FRE.HN	-0,3677	0,012664
IV.ERBM.EH	-0,92324	0,015279
IT.FOR.HN	0,39205	0,013132
IT.VDB.HG	0,28978	0,36184
IT.VALS.HN	0,05754	0,10991
IT.DANT.HN	0,25275	0,01243
IT.SLOB.HN	-0,027745	0,015461
IT.BNO.HN	-0,51426	0,11006
IT.APR.HG	-0,31877	0,11494
IT.SGVC.HN	0,97919	0,37721
IT.BRA.HG	0,24985	0,12233
IV.SAL.EH	-0,57901	0,46928
IV.BOZZ.HH	0,42253	0,014164
IT.DSG.HG	0,11951	0,25172
IT.MLBT.HN	0,062972	0,061478
IT.SON.HG	0,098107	0,093403
IT.SEM.HG	0,10059	0,19661
IT.RST.HN	-0,51691	0,12331

IT.BGMO.HN	0,27509	0,12432
IT.FLP.HG	1,0287	0,46529
IT.GRA.HN	0,91569	0,46541
IT.TRTO.HN	0,08958	0,030267
IT.BLZN.HN	0,47411	0,011732
IT.MRNO.HN	0,73659	0,32294
IT.PNAL.HN	1,1269	0,014845
IT.BRSA.HG	0,014552	0,012405
IT.LEC.HN	-0,15664	0,012478

Table S2. Coefficients of the attenuation models for PDs (C in Eq.1).

Dist. (km)	C (Eq. 1)
2,0	-1,684
5,0	-1,528
5,4	-1,372
5,8	-1,216
6,2	-1,060
6,6	-0,904
7,1	-0,748
7,6	-0,592
8,2	-0,436
8,8	-0,280
9,4	-0,124
10,1	0,032
10,9	0,003
11,7	-0,032
12,5	-0,009
13,5	-0,134
14,4	-0,094
15,5	-0,262
16,6	-0,266
17,9	-0,355
19,2	-0,395
20,6	-0,467
22,1	-0,538
23,7	-0,605
25,4	-0,690
27,3	-0,789
29,3	-0,857
31,4	-0,871
33,7	-0,932
36,2	-1,010
38,9	-1,079
41,7	-1,115
44,8	-1,196
48,1	-1,212
51,6	-1,297
55,4	-1,324
59,4	-1,387
63,8	-1,447
68,4	-1,453

73,5	-1,504
78,8	-1,538
84,6	-1,557
90,8	-1,604
97,5	-1,610
104,6	-1,619
112,3	-1,666
120,5	-1,671
129,3	-1,676
138,8	-1,682
149,0	-1,687

Table S3. b-value estimates for the grid nodes in southeastern Alps.

ID grid node	b+	sb+	latitude	longitude	N. Eqs.
1	-0,90	0,04	10,5792	45,8195	242
2	-1,11	0,06	10,5792	45,9993	234
3	-0,81	0,04	10,7591	45,6396	272
4	-0,86	0,03	10,7591	45,8195	431
5	-1,03	0,05	10,7591	45,9993	253
6	-0,83	0,03	10,9389	45,6396	376
7	-0,92	0,03	10,9389	45,8195	472
8	-1,08	0,05	10,9389	45,9993	316
9	-1,35	0,08	10,9389	46,1792	201
10	-0,95	0,04	11,1188	45,6396	346
11	-0,96	0,03	11,1188	45,8195	446
12	-1,07	0,04	11,1188	45,9993	395
13	-1,06	0,05	11,2986	45,6396	235
14	-0,98	0,04	11,2986	45,8195	348
15	-1,23	0,06	11,2986	45,9993	296
16	-1,02	0,06	12,0181	45,9993	217
17	-1,02	0,06	12,198	45,9993	274
18	-0,93	0,03	12,198	46,1792	361
19	-1,03	0,05	12,3778	45,9993	306
20	-0,99	0,03	12,3778	46,1792	517
21	-0,88	0,03	12,3778	46,3591	575
22	-1,08	0,06	12,5577	45,9993	273
23	-0,89	0,03	12,5577	46,1792	658
24	-0,88	0,02	12,5577	46,3591	748
25	-0,84	0,03	12,5577	46,5389	331
26	-0,90	0,02	12,7376	46,1792	665
27	-0,87	0,02	12,7376	46,3591	1075
28	-0,84	0,02	12,7376	46,5389	600
29	-0,95	0,03	12,9174	46,1792	712
30	-0,89	0,02	12,9174	46,3591	1432
31	-0,92	0,02	12,9174	46,5389	840
32	-1,01	0,03	13,0973	46,1792	856
33	-0,93	0,02	13,0973	46,3591	1384
34	-0,88	0,02	13,0973	46,5389	868
35	-0,98	0,03	13,2772	46,1792	831
36	-0,95	0,02	13,2772	46,3591	1251
37	-0,90	0,02	13,2772	46,5389	642
38	-0,95	0,04	13,457	45,9993	323
39	-0,92	0,03	13,457	46,1792	768

40	-0,90	0,02	13,457	46,3591	910
41	-0,99	0,04	13,457	46,5389	351
42	-0,99	0,05	13,6369	45,9993	341
43	-0,91	0,02	13,6369	46,1792	651
44	-0,92	0,03	13,6369	46,3591	535
45	-1,04	0,04	13,6369	46,5389	333
46	-1,17	0,07	13,8167	45,8195	202
47	-1,15	0,05	13,8167	45,9993	277
48	-0,92	0,04	13,8167	46,1792	487
49	-0,87	0,04	13,8167	46,3591	378
50	-1,17	0,06	13,8167	46,5389	204
51	-1,09	0,04	13,9966	45,6396	242
52	-1,10	0,05	13,9966	45,8195	251
53	-1,06	0,05	13,9966	45,9993	265
54	-0,94	0,04	13,9966	46,1792	248
55	-0,97	0,05	14,1765	45,6396	269
56	-1,04	0,05	14,1765	45,8195	272
57	-1,12	0,05	14,1765	45,9993	319
58	-1,04	0,05	14,1765	46,1792	311
59	-0,97	0,05	14,3563	45,6396	225
60	-0,98	0,05	14,3563	45,8195	249
61	-1,05	0,05	14,3563	45,9993	294
62	-1,10	0,05	14,3563	46,1792	295

Table S4. b-value for the first (b_0) and the last (b_f) years for each grid node.

ID grid node	latitude	longitude	b0	bf	T0 (yy)	T0 (mm)	T0 (dd)	Tf (yy)	Tf (mm)	Tf (dd)
1	10,7591	45,8195	-0,90	-1,01	2020	5	24	2025	2	27
2	10,9389	45,6396	-0,84	-0,99	2020	11	11	2025	2	27
3	10,9389	45,8195	-0,93	-1,10	2020	5	6	2025	2	27
4	10,9389	45,9993	-1,15	-1,25	2022	9	28	2025	2	24
5	11,1188	45,6396	-0,99	-1,05	2020	12	23	2025	2	24
6	11,1188	45,8195	-1,03	-1,13	2020	5	31	2025	2	24
7	11,1188	45,9993	-1,32	-1,34	2022	6	8	2025	2	24
8	11,2986	45,8195	-1,01	-1,08	2021	3	2	2025	2	24
9	12,198	46,1792	-0,90	-1,04	2020	4	21	2025	2	7
10	12,3778	45,9993	-1,05	-1,08	2021	8	3	2025	2	5
11	12,3778	46,1792	-1,09	-0,98	2019	1	28	2025	2	16
12	12,3778	46,3591	-0,93	-0,85	2018	9	29	2025	2	16
13	12,5577	46,1792	-1,01	-0,82	2018	8	24	2025	2	16
14	12,5577	46,3591	-1,00	-0,82	2018	8	29	2025	2	26
15	12,5577	46,5389	-0,89	-0,78	2021	5	27	2025	2	26
16	12,7376	46,1792	-1,06	-0,78	2018	12	31	2025	2	23
17	12,7376	46,3591	-1,00	-0,85	2018	2	11	2025	2	26
18	12,7376	46,5389	-0,89	-0,80	2019	6	28	2025	2	26
19	12,9174	46,1792	-1,04	-0,91	2019	3	4	2025	2	23
20	12,9174	46,3591	-0,99	-0,91	2017	9	27	2025	2	27
21	12,9174	46,5389	-0,99	-0,94	2018	6	16	2025	2	26
22	13,0973	46,1792	-1,05	-1,06	2018	8	25	2025	2	23
23	13,0973	46,3591	-1,05	-0,94	2017	10	1	2025	2	27
24	13,0973	46,5389	-1,00	-0,82	2018	6	10	2025	2	27
25	13,2772	46,1792	-1,03	-0,97	2018	10	13	2025	2	21
26	13,2772	46,3591	-1,01	-0,96	2017	11	3	2025	2	27
27	13,2772	46,5389	-1,02	-0,81	2018	12	17	2025	2	27
28	13,457	45,9993	-0,97	-1,00	2022	9	5	2025	2	27
29	13,457	46,1792	-0,94	-0,94	2018	12	4	2025	2	27
30	13,457	46,3591	-0,97	-0,87	2018	6	23	2025	2	27
31	13,457	46,5389	-0,94	-0,99	2021	12	5	2025	2	27
32	13,6369	45,9993	-1,05	-1,01	2022	3	22	2025	2	27
33	13,6369	46,1792	-0,94	-0,88	2019	6	13	2025	2	27
34	13,6369	46,3591	-0,92	-0,96	2019	10	20	2025	2	23
35	13,6369	46,5389	-0,93	-1,21	2022	1	26	2025	2	25
36	13,8167	46,1792	-0,96	-0,98	2020	3	9	2025	2	27
37	13,8167	46,3591	-0,84	-0,92	2021	3	7	2025	2	25
38	14,1765	45,9993	-1,21	-1,40	2022	10	29	2025	2	11
39	14,1765	46,1792	-1,15	-1,37	2022	10	2	2025	1	30
